# Supplementary material for: Turning the tide: a quasi-experimental study on a coaching intervention to reduce burn-out symptoms and foster personal resources among medical residents and specialists in the Netherlands
Source: BMJ Open. 2021 Jan 25;11(1):e041708. doi: 10.1136/bmjopen-2020-041708 (PMC7839886; doi:10.1136/bmjopen-2020-041708)
Supplement: Author's manuscript [file bmjopen-2020-041708.draft_revisions.pdf]

BMJ Open is committed to open peer review. As part of this commitment we make the peer review history of every article we publish publicly available.

When an article is published we post the peer reviewers' comments and the authors' responses online. We also post the versions of the paper that were used during peer review. These are the versions that the peer review comments apply to.

The versions of the paper that follow are the versions that were submitted during the peer review process. They are not the versions of record or the final published versions. They should not be cited or distributed as the published version of this manuscript.

BMJ Open is an open access journal and the full, final, typeset and author-corrected version of record of the manuscript is available on our site with no access controls, subscription charges or pay-per-view fees (http://bmjopen.bmj.com).

If you have any questions on BMJ Open's open peer review process please email info.bmjopen@bmj.com

BMJ Open

Turning the tide: a controlled coaching intervention to reduce burnout symptoms and foster personal resources among medical residents and specialists in the Netherlands

Journal:	*BMJ Open*
Manuscript ID	bmjopen-2020-041708
Article Type:	Original research
Date Submitted by the Author:	15-Jun-2020
Complete List of Authors:	Solms, Lara; University of Amsterdam, Work and Organizational Psychology; Erasmus Medical Center - Sophia Children's Hospital, Pediatrics<br>van Vianen, Annelies; University of Amsterdam, Work and Organizational Psychology<br>Koen, Jessie; University of Amsterdam, Work and Organizational Psychology<br>Theeboom, Tim; Vrije Universiteit Amsterdam, School of Business and Economics<br>de Pagter, Anne; Erasmus Medical Center - Sophia Children's Hospital , Pediatric Hematology; Leiden University Medical Center, Pediatrics<br>De Hoog, Matthijs; Erasmus Medical Center - Sophia Children's Hospital, Pediatrics/ Pediatric Intensive Care Unit
Keywords:	EDUCATION & TRAINING (see Medical Education & Training), Human resource management < HEALTH SERVICES ADMINISTRATION & MANAGEMENT, MENTAL HEALTH

SCHOLARONE™
Manuscripts

I, the Submitting Author has the right to grant and does grant on behalf of all authors of the Work (as defined in the below author licence), an exclusive licence and/or a non-exclusive licence for contributions from authors who are: i) UK Crown employees; ii) where BMJ has agreed a CC-BY licence shall apply, and/or iii) in accordance with the terms applicable for US Federal Government officers or employees acting as part of their official duties; on a worldwide, perpetual, irrevocable, royalty-free basis to BMJ Publishing Group Ltd ("BMJ") its licensees and where the relevant Journal is co-owned by BMJ to the co-owners of the Journal, to publish the Work in this journal and any other BMJ products and to exploit all rights, as set out in our licence.

The Submitting Author accepts and understands that any supply made under these terms is made by BMJ to the Submitting Author unless you are acting as an employee on behalf of your employer or a postgraduate student of an affiliated institution which is paying any applicable article publishing charge ("APC") for Open Access articles. Where the Submitting Author wishes to make the Work available on an Open Access basis (and intends to pay the relevant APC), the terms of reuse of such Open Access shall be governed by a Creative Commons licence – details of these licences and which Creative Commons licence will apply to this Work are set out in our licence referred to above.

Other than as permitted in any relevant BMJ Author's Self Archiving Policies, I confirm this Work has not been accepted for publication elsewhere, is not being considered for publication elsewhere and does not duplicate material already published. I confirm all authors consent to publication of this Work and authorise the granting of this licence.

For peer review only

Turning the tide: a controlled coaching intervention to reduce burnout symptoms and foster personal resources among medical residents and specialists in the Netherlands

Lara Solms, MSc, Annelies E.M. van Vianen, PhD, Jessie Koen, PhD, Tim Theeboom, PhD, Anne P.J. de Pagter*, MD, PhD, Matthijs de Hoog*, MD, PhD on behalf of the Challenge & Support Research Network

L. Solms is PhD candidate, Department of Work and Organizational Psychology, University of Amsterdam, Amsterdam, The Netherlands, PhD candidate, Department of Pediatrics, Erasmus MC-Sophia Children's Hospital, Rotterdam, The Netherlands

A.E.M van Vianen is professor of work and organizational psychology, Department of Work and Organizational Psychology, University of Amsterdam, Amsterdam, The Netherlands

T. Theeboom is assistant professor, School of Business and Economics, Vrije Universiteit Amsterdam, Amsterdam, The Netherlands

J. Koen is assistant professor, Department of Work and Organizational Psychology, University of Amsterdam, Amsterdam, The Netherlands

A.P.J. de Pagter is a pediatrician-hematologist, Department of Pediatrics, Erasmus MC-Sophia Children's Hospital, Rotterdam, The Netherlands

M. de Hoog is pediatric intensivist, professor of postgraduate medical education, director of pediatric residency training & director of PICU, Erasmus MC-Sophia Children's Hospital, Rotterdam, The Netherlands

The Challenge & Support Research Network:

Annemarie M.C. van Rossum, Wouter J.W. Kollen, Robbert G.M. Bredius, Amando J. Heesterman, Marlies A. van Houten, Marie-José E. Walenkamp, Adrienne A.M. Zandbergen, Stephanie C.E. Schuit, Jacoline E.C. Bromberg, Axel Willemse, Sarah M. van den Hee, Machteld van den Heuvel, and Angelique Bakker-Pieper.

For peer review only - http://bmjopen.bmj.com/site/about/guidelines.xhtml

*A.P.J. de Pagter and M. de Hoog contributed equally to this paper.

Correspondence should be addressed to Anne P.J. de Pagter, Department of Pediatrics, Erasmus MC-Sophia Children’s Hospital, Rotterdam, Sp-2430. Wytemaweg 80, 3015 CN Rotterdam, The Netherlands; telephone: +31107032435; e-mail: p.depagter@erasmusmc.nl

Manuscript word count: 3656

Keywords: Coaching; professional development; burnout; personal resources; residents; specialists

For peer review only

For peer review only - http://bmjopen.bmj.com/site/about/guidelines.xhtml

ABSTRACT

Objectives

Physician burnout is increasing, starting already among residents. The consequences of burnout are not limited to physicians' well-being, they also pose a threat to patient care and safety. This study investigated the effectiveness of a professional coaching intervention to reduce burnout symptoms and foster personal resources in residents and specialists.

Design

In a controlled field experiment, medical residents and specialists received six professional coaching sessions, while a control group did not undergo any treatment. The authors assessed burnout symptoms of exhaustion and cynicism, the personal resources psychological capital, psychological flexibility, and self-compassion, as well as job demands and job resources with validated questionnaires (January 2017 until August 2018). The authors conducted repeated measures analyses of variance (ANOVA) procedures to examine changes over time for the intervention and the control group.

Setting

Four academic hospitals in the Netherlands.

Participants

A final sample of 57 residents and specialists volunteered in an individual coaching program. A control group of 57 physicians did not undergo any treatment.

Intervention

Coaching was provided by professional coaches during a period of approximately 10 months aiming at personal development and growth.

For peer review only

Results

The coaching group (68%, 57 physicians, 10 men, 47 women) reported a reduction in burnout symptoms and an increase in personal resources after the coaching intervention, while no such changes occurred in the control group (35 %, 15 men, 42 women), as indicated by significant Time x Group interactions, all p's < .01. Specifically, physicians increased their psychological capital (η_p^{2} = .139), their self-compassion (η_p^2=.083), and reported significantly less exhaustion (η_p^2=.126), the main component of the burnout syndrome.

Conclusion

This study suggests that individual coaching is a promising route to reduce burnout symptoms in both residents and specialists. Moreover, it strengthens personal resources that play a crucial role in the prevention of burnout.

Article summary

Strengths and limitations of this study

- This study provides first evidence from a controlled intervention study on the effectiveness of coaching in both medical residents and specialists.
- Six individual professional face-to-face coaching sessions can decrease burnout symptoms (i.e., exhaustion) among medical residents and specialists.
- Preventive coaching contributes to the improvement of the personal resources psychological capital and self-compassion, resources that play a role in the prevention of burnout.
- The study is limited by its quasi-experimental design. However, the analyses controlled for initial differences between the coaching and the control group.
- The coaching group consisted exclusively of pediatric residents and physicians. Consequently, more research is needed that evaluates the effectiveness of coaching in

For peer review only

different specialties, allowing broader generalization for coaching effectiveness among healthcare professionals.

For peer review only

For peer review only - http://bmjopen.bmj.com/site/about/guidelines.xhtml

INTRODUCTION

Physicians experience a variety of stressors including time pressure, emotionally taxing patient-interactions, and an increasing bureaucratic burden. Not surprisingly, burnout (i.e., feeling exhausted, dissociated and less efficient) is high among senior healthcare professionals as well as residents [1-2]. Burnout has severe consequences for physicians, often leading to long-term absenteeism and eventually abandonment of the medical profession[3]. But the negative consequences are not limited to physicians' well-being and careers: with burnout flooding the healthcare system, patient safety is also at risk. Physician burnout is associated with a twofold increase in patient safety incidents as well as poorer quality of care due to low professionalism[4].

In order to combat burnout among physicians, and thus warrant patient safety, powerful interventions are needed that put physicians' needs first. This is the case in professional coaching in which the coach "facilitates the enhancement of life experience and goal-attainment in the personal and/or professional life of normal, non-clinical clients"[5]. Surprisingly, coaching is not common in medical practice and research is scarce[6-10] despite the fact that the positive effects of coaching on well-being and functioning have been demonstrated in a number of educational and professional settings[11]. Furthermore, with coaching being generally connected to problem elimination (e.g., burnout) in healthcare, rather than to professional development and well-being, its power is underrated if not invisible due to stigma. Given the potential benefits of coaching for physician well-being, research on the effectiveness of coaching in a professional development setting is sorely needed.

A professional coaching intervention may simultaneously help to resolve and prevent burnout among physicians. That is, professional coaching can not only directly reduce

For peer review only

For peer review only - http://bmjopen.bmj.com/site/about/guidelines.xhtml

burnout symptoms, but can also strengthen personal resources that may prevent such burnout symptoms in the first place[12]. This assumption is rooted in research on burnout, which shows that the onset of burnout is caused by both heavy job demands and a lack of (personal) resources[13]. According to the Job Demands-Resources Model (JD-R)[13], a common work-stress model in the prediction of burnout and work engagement, personal resources help people to deal with extreme demands, ultimately buffering the negative effects of job demands on burnout[14]. At the same time, personal resources stimulate motivation and work engagement. With both work engagement and well-being (i.e., a lack of burnout) being indispensable for optimal physician functioning, the value of professional coaching lies in its ability to kill two birds with one stone: It aims to reduce burnout symptoms as well as stimulate life-long reflection and self-management through recognizing and strengthening individuals' personal resources.

In this two-wave quasi-experimental study we evaluated the benefits of an individual coaching program for the resources, demands, and well-being (i.e., lack of burnout symptoms), and work engagement of medical residents and specialists in the Netherlands.

METHOD

Study Setting and Population

This study evaluates the effectiveness of an individual coaching program in two major academic hospitals, the Erasmus Medical Center (EMC) and the Leiden University Medical Center (LUMC) in the Netherlands. A final number of 114 physicians participated in this study of which 57 received individual coaching between January 2017 and August 2018. The coaching program was completely voluntary, offering six individual coaching sessions to both residents and specialists from the pediatrics department at the EMC and LUMC. Because funding for the coaching program was initially only available for the pediatrics department, physicians from other departments (i.e., internal medicine, neurology) and

For peer review only - http://bmjopen.bmj.com/site/about/guidelines.xhtml

pediatric residents from two other hospitals served as a control group. Additionally, pediatricians who did not voice interest in the coaching program were placed in the control group as well. See Table 1 for sample characteristics. The study protocol was approved by the institutional ethics board at the University of Amsterdam.

Intervention and Procedure

Physicians were informed through different channels (i.e., e-mail newsletter, information presentation, mouth-to-mouth) about the coaching program and could sign themselves up for the program via e-mail. Physicians that voiced interest in the coaching program, were asked to participate in the study and were able to choose a coach of their preference. All coaches participating in the program were selected based on a number of relevant criteria such as years of experience and affinity and experience with the medical profession. Physicians could view introductory videoclips of coaches on the program website. In these 1-minute long videos, coaches introduced themselves and gave information about their way of working with clients. Thereafter, physicians chose their coach and the first coaching session was arranged. All participants started their coaching trajectory individually depending on the availability of their coach. Before the coaching started, informed consent was obtained from all participants. Demographics as well as the study variables were measured with an online survey delivered via Qualtrics (Qualtrics, 2005) shortly before the first coaching session at baseline (T1) and minimal 7 days (M = 87.25, SD = 92.95, range: 7-364) after the last coaching session was finished (T2). Participants that failed to fill out the T1 or T2 survey at first, received up to three reminders by e-mail with the request to complete the survey. For a description of exclusion criteria, see Figure 1.

Study Variables

In line with the JD-R model, we measured job demands (workload, job insecurity, work-family conflict), job resources (autonomy, colleague support, supervisor support), personal

For peer review only - http://bmjopen.bmj.com/site/about/guidelines.xhtml

resources (psychological capital, self-compassion, psychological flexibility), as well as burnout symptoms and work engagement.

Job demands. We measured workload, job insecurity, and work-family conflict.

Workload was assessed with four items from the Quantitative Workload Inventory[15] and two additional items that were added to match the specific demands of medical practice. The two additional items assessed working overtime and emotional strain. All items were measured on a 5-point scale ranging from 1 ("never") to 5 ("always").

Job insecurity, that is, "the perceived threat of job loss and the worries related to that threat" was assessed with a 5-item adapted version of the Job Insecurity Scale[16]. The items were scored on a 7-point scale ranging from 1 ("not at all applicable") to 7 ("very applicable").

Work-Family Conflict was measured with four items of the Work-Family Conflict Scale[17] assessing "the general demands of, time devoted to, and strain created by the work interfere with performing family-related responsibilities". The items were scored on a 7-point scale ranging from 1 ("not at all applicable") to 7 ("very applicable").

Job resources. Job resources encompassed autonomy, supervisor support, and colleague support.

Autonomy was measured with nine items from the Work Design Questionnaire[18] assessing perceived autonomy with regard to work scheduling and methods, and decision-making. The items were scored on a seven-point scale ranging from 1 ("totally disagree") to 7 ("totally agree").

Supervisor support, that is, the experienced psychological and work support from the supervisor was assessed with six items from Vinokur, Schul, and Caplan[19]. For residents, supervisory support measured the support received from the training supervisor, whereas for specialists, supervisory support measured the support received from the head of the

For peer review only - http://bmjopen.bmj.com/site/about/guidelines.xhtml

department. The items were scored on a seven-point scale ranging from 1 (“totally disagree”) to 7 (“totally agree”).

Colleague support, the experienced psychological and work support from colleagues, was assessed with the same six items as supervisor support[19], but the items referred to colleagues instead of the supervisor.

Personal resources. We measured psychological capital, self-compassion, and psychological flexibility.

To capture P*sychological capital*’s components, hope, optimism, and resilience, we used 9 items from the Dutch version of the PsyCap questionnaire[20]. To measure the fourth component, self-efficacy, we used 3 items based on the Generalized Self-efficacy scale[21] that were adapted so they would fit the occupational setting as used in previous research[22]. The items were scored on a seven-point scale ranging from 1 (“totally disagree”) to 7 (“totally agree”).

Self-compassion, that is “treating oneself with kindness, recognizing one’s shared humanity, and being mindful when considering negative aspects of oneself” was measured with six items from the Self-Compassion Scale[23]. This scale encompasses three subscales: self-kindness, common humanity and mindfulness. The items were scored on a five-point scale ranging from 1 (“rarely”) to 5 (“almost always”).

Psychological flexibility, that is, the ability to flexibly take appropriate action towards achieving goals and values, even in the presence of challenging or unwanted events was measured with seven items of the Work Acceptance and Action Questionnaire[24]. The items were scored on a five-point scale ranging from 1 (“rarely”) to 5 (“almost always”).

Burnout symptoms and work engagement.

Burnout symptoms. We measured burnout symptoms with the two core scales exhaustion and cynicism of the Dutch version of the Maslach Burnout Inventory[25-26]. Both scales were

For peer review only

measured with 5 and 4 items respectively. The items were scored on a seven-point scale ranging from 1 (“totally disagree”) to 7 (“totally agree”).

Work engagement. We measured work engagement with the Utrecht Work Engagement Scale[27]. It’s nine items cover the three subscales vigor, dedication, and absorption. The items were scored on a seven-point scale ranging from 1 (“never”) to 7 (“always”).

Statistical Analyses

Intervention effects. To test if the coaching intervention would have beneficial effects, repeated measures analyses of variance (ANOVA) procedures were performed to examine changes over time for the intervention and the control group. The outcomes analyzed were job demands (workload, job insecurity, work-family conflict), job resources (autonomy, colleague support, supervisor support), personal resources (psychological capital self-compassion, psychological flexibility), as well as burnout symptoms (exhaustion, cynicism) and work engagement. We controlled for coaching attitude, i.e., the degree to which one believes coaching is beneficial or helpful, because it can be expected that a positive attitude may contribute to the intervention success. Significant Time x Group interactions of the outcome variables were followed up with post hoc tests.

Preliminary analyses. ***Self-selection of participants***. Because participation in the coaching program was voluntarily – and complete randomization of participants to conditions was therefore not feasible - we examined structural demographic differences prior to the intervention between the coaching and the control group (T1). These demographics were gender, age, tenure (i.e., medical resident, specialist), department (i.e., pediatrics, internal medicine, neurology) and hospital affiliation (i.e., EMC, LUMC, VUMC, AMC). Sample characteristics are displayed in Table 1. While both groups did not differ with respect to gender ($x^2(1) = 1.28$, $p = .26$), age ($F\,(1{,}112) = 0.49$, $p = .49$), and tenure ($x^2(1) = 0.33$, $p = .57$), they did differ in department affiliation ($x^2(2) = 32.02$, $p < .001$) and hospital affiliation

For peer review only - http://bmjopen.bmj.com/site/about/guidelines.xhtml

($x^2(3) = 22.55$, $p < .001$). More specifically, all coaching participants were affiliated with the pediatrics department of two of the four participating hospitals. We conducted three types of additional analyses to rule out that potential effects attributed to the coaching intervention were caused by factors related to the imbalance of department and hospital affiliation– although conceptually, this is highly unlikely. *Hospital affiliation.* To estimate a potential impact of hospital affiliation on treatment effectiveness, we conducted multiple univariate repeated measures for each of the outcome variables including hospital affiliation as additional control variable to see if the previous results would hold. Additionally, we conducted the original analyses solely for physicians employed at the two medical hospitals that were represented in the intervention group. *Department affiliation.* Given that all participants in the coaching intervention were affiliated with the pediatrics department we analyzed whether pediatricians differed from physicians affiliated with other departments (e.g., neurology, internal medicine) with respect to contextual variables, here c*ompetition*, and *psychological safety,* variables that reflect experienced department work climate and potentially could influence treatment effectiveness.

Table 1. Characteristics of the Study Population in a Study on Coaching Effectiveness for Medical Residents and Specialists, 2017-2018[a]

**Characteristics**	**Intervention (N = 57)**	**Control (N = 57)**
Male sex – no. (%)	10 (17.5)	15 (26.3)
**Age - yr**		
Median	33	35
Interquartile range	9.5	12
**Specialty – no (%)**		
Pediatrics	57 (100)	32 (56.1)
Internal medicine	-	15 (26.3)
Neurology	-	10 (17.5)
**Professional role – no (%)**		

For peer review only - http://bmjopen.bmj.com/site/about/guidelines.xhtml

Resident	33 (57.9)	36 (63.2)
Specialist	24 (42.1)	21 (36.8)
**Hospital – no (%)**		
EMC	32 (56.1)	33 (57.9)
LUMC	25 (43.9)	9 (15.8)
VUMC	-	7 (12.3)
AMC	-	8 (14.0)
Coaching experience – no. (%)	22 (38.6)	19 (33.3)
**Home situation – no (%)**		
Children, one or more	28 (49.1)	29 (50.9)
No children	29 (50.9)	28 (49.1)

[a]This study was conducted at 4 academic hospitals in the Netherlands. In this study, the authors investigated the effects of an individual coaching intervention on burnout symptoms, work engagement, personal resources, job demands and job resources among pediatric residents and specialists.

Baseline differences between groups. With respect to the outcome variables at baseline, we found significant differences between the intervention and the control group: The intervention group scored significantly lower on personal resources, and significantly higher on job demands and exhaustion, similar to the results of a previous study on counseling in Norwegian doctors[28]. An overview of the differences between the groups is displayed in Table 2. Because distribution of participants was not random, and because there were significant differences on a number of outcomes prior to the intervention, we tested our hypotheses with repeated measures analysis of variance. These analyses are favored over the analysis of covariance in a non-randomized intervention study[29]. Additionally, we followed the recommendations of Huberty and Moris[30] and conducted multiple ANOVAs as opposed to a MANOVA as a preliminary step to multiple ANOVAs.

Table 2. Means and Standard Deviations (*SD*) of Study Variables for the Control and the Intervention Group at Baseline (T1) in a Study on Coaching Effectiveness for Medical Residents and Specialists, 2017-2018[a]

**Study Variables**	**Intervention (N = 57)**	**Control (N = 57)**
	Mean (*SD*)	Mean (*SD*)

For peer review only - http://bmjopen.bmj.com/site/about/guidelines.xhtml

**Job demands**		
Workload**	3.48 (.67)	3.10 (.78)
Job insecurity**	4.24 (1.33)	3.37 (1.45)
Work-family conflict**	4.85 (1.05)	4.00 (1.19)
**Job resources**		
Autonomy	4.39 (1.03)	4.67 (1.14)
Colleague support	5.33 (.96)	5.47 (.90)
Supervisor support	4.63 (1.51)	4.98 (1.42)
**Personal resources**		
PsyCap**	4.83 (.69)	5.19 (.72)
Self-compassion**	3.07 (.60)	3.39 (.66)
Psych. flexibility*	3.43 (.63)	3.67 (.53)
**Outcomes**		
Exhaustion**	2.75 (1.08)	2.13 (.92)
Cynicism	2.11 (1.08)	2.06 (.93)
Work engagement	5.08 (.78)	5.04 (.75)

Abbreviation: SD indicates standard deviation; PsyCap indicates psychological capital; psych. flexibility indicates psychological flexibility.
[a]Differences in means between the intervention and the control group are indicated by the following significance values: $^{\dagger}p < .10$; $^{*}p < .05$; $^{**}p < .01$.

Patient and public involvement

This study investigated the effectiveness of a professional coaching intervention in medical residents and specialists. No patients or public representatives were involved in the study.

RESULTS

A total number of 84 physicians signed up for the coaching program while 161 physicians signed up for the control group. Of these two groups, 57 physicians in each group completed the follow-up measurement and were included in the final sample (Figure 1). Table 1 shows the demographic characteristics of the study population. Internal consistencies ranged from .75 to .95 and were acceptable for all scales. See Table 4 for correlations between the study variables at baseline.

For peer review only

Intervention effects. The analyses revealed significant changes in the intervention group that did not occur in the control group, as indicated by significant Group x Time interactions for a number of outcomes. A summary of the results is shown in Table 3. With regard to job demands, post hoc analyses revealed a decrease in job insecurity and work-family conflict in the intervention group with both p's < .05. With regard to job resources, post-hoc analyses showed that autonomy increased in the intervention group, while supervisor support decreased in the control group, all p's < .05. With regard to personal resources, post hoc comparisons indicated an increase in psychological capital and self-compassion in the intervention group, all p's < .05, as well as a decrease in self-compassion in the control group, $p < .05$. No changes occurred in psychological flexibility, in either the control or coaching group, all p's >.05. Finally, with regard to outcomes, analyses showed that the coaching group significantly decreased their burnout symptoms but showed no changes in work engagement. Post hoc comparisons indicated a decrease in exhaustion in the intervention group, $p < .05$, while no such changes occurred in the control group, all p's > .05 or with regard to cynicism, $p > .05$. For a graphical representation of these effects, see Figure 2.

For peer review only

Table 3. Summary of Results for Repeated Measures Analyses and Pre- and Postintervention Means for the Intervention Group in a Study on Coaching Effectiveness for Medical Residents and Specialists, 2017-2018[a-c]

Time x Group interaction for study variables	Mean Square	*F*	*p*	η_p^2	Preintervention Mean (*SD*)	Postintervention Mean (*SD*)	*Df*	*t*	*p*
**Job demands**									
Workload	.211	.837	.362	.007	3.48 (.67)	3.31 (.61)	56	1.97	.053
Job insecurity**	6.07	10.99	.001	.090	4.24 (1.33)	3.61 (1.46)	56	4.10	.000
Work- family-conflict**	4.60	8.33	.005	.070	4.85 (1.05)	4.34 (1.12)	56	4.36	.000
**Job resources**									
Autonomy**	3.41	7.56	.007	.064	4.39 (1.03)	4.89 (1.06)	56	-4.19	.000
Colleague support*	1.68	4.68	.033	.040	5.33 (.96)	5.56 (.79)	56	-1.94	.057
Supervisor support*	3.79	5.60	.020	.048	4.63 (1.51)	4.82 (1.35)	56	-1.28	.207
**Personal resources**									
PsyCap**	2.57	17.92	.000	.139	4.83 (.69)	5.16 (.65)	56	-4.08	.000
Self-compassion**	1.26	10.00	.002	.083	3.07 (.60)	3.27 (.52)	56	-2.72	.009
Psych. flexibility	.335	1.80	.182	.016	3.43 (.63)	3.47 (.65)	56	-0.53	.600
**Outcomes**									
Exhaustion**	6.20	15.94	.000	.126	2.75 (1.08)	2.25 (.79)	56	4.00	.000
Cynicism*	2.52	5.44	.022	.047	2.11 (1.08)	1.90 (.75)	56	1.46	.151
Work engagement†	.69	3.19	.077	.028	5.08 (.78)	5.28 (.59)	56	-2.19	.033

[a]The following significance values are used: †$p < .10$; *$p < .05$; **$p < .01$.

[b]η_p^2 refers to the degree to which variability among observations can be attributed to conditions controlling for the subjects' effect that is unaccounted for by the model.

[c]*Df* for the time x group interaction = 1 for all study variables and 111 for the error(time)

For peer review only

Table 4. Correlations between the Study Variables for the Intervention and Control Group* at Baseline in a Study on Coaching Effectiveness for Medical Residents and Specialists, 2017-2018[a]

Study Variables	1	2	3	4	5	6	7	8	9	10	11	12	13
1. Coaching attitude	1	.284*	-.032	.015	-.024	-.088	-.094	-.218	-.203	.172	-.014	.006	.092
2. Workload	-.050	1	-.089	.322*	.015	-.124	-.209	-.017	-.067	.12	.299*	.168	-.066
3. Job insecurity	.128	.191	1	-.066	-.458**	-.278*	.095	-.511**	-.405**	-.220	.225	.221	-.285*
4. Work-family conflict	-.142	.454**	-.071	1	.038	-.045	.131	.060	-.107	-.004	.341**	.207	-.100
5. Autonomy	-.025	-.333	-.335	-.232	1	.161	-.036	.382**	.224	.142	-.177	-.141	.290*
6. Colleague support	.234	-.163	-.153	-.040	.348	1	-.100	.418**	.348**	.137	-.463**	-.510**	.557**
7. Supervisor support	-.026	.020	-.379	-.035	.341*	.233	1	.154	-.031	-.076	.140	-.032	.027
8. PsyCap	-.173	-.370	-.463	-.199	.486**	.325*	.401**	1	.607**	.242	-.365**	-.576**	.627**
9. Self-compassion	-.176	-.276	-.422	-.260	.244	.362**	.427**	.512**	1	.086	-.545**	-.397**	.388**
10. Psych. flexibility	-.238	.031	-.170	.189	.415**	.256	.212	.273*	.209	1	-.094	-.187	.407**
11. Exhaustion	.064	.493**	.313*	.411**	-.299*	-.222	-.318*	-.363**	-.439**	-.157	1	.602**	-.570**
12. Cynicism	-.111	.355**	.356**	.005	-.339**	-.328*	-.373**	-473**	-.286*	-.337	.686**	1	-.617**
13. Work engagement	.228	-.164	-.228	-.041	.309*	.349**	.491**	.451**	.303*	.403**	-.482**	-.712	1

Abbreviation: PsyCap indicates psychological capital; psych. flexibility indicates psychological flexibility.
[a]Above the diagonal: coaching group; below the diagonal: control group

.

For peer review only - http://bmjopen.bmj.com/site/about/guidelines.xhtml

Supplementary analyses

In order to rule out that effects attributed to the intervention were (partly) influenced by hospital and department affiliation we conducted three additional analyses[1].

Hospital affiliation. First, we conducted repeated measures analyses for each outcome variable with the whole sample, but this time added hospital affiliation as a control variable. The results of these analyses revealed no significant differences with those of the original analyses, except for work engagement as outcome. Here, we find (instead of a marginal significant) a significant group x time interaction, p = .019. Post hoc analyses indicated that the coaching group reported increased work engagement after the coaching program, with no changes occurring in the control group. Overall, these results indicate that hospital affiliation did not influence treatment effectiveness in significant ways. Additionally, we examined whether the results of the whole sample (including four hospitals) were comparable to those of a subsample including only physicians from the two academic hospitals that offered the coaching intervention (EMC and LUMC). We conducted repeated measures analyses for each outcome variable. Coaching attitude, i.e., the degree to which one believes coaching is beneficial or helpful, was included in the analyses as control variable. The results of the analyses with the subsample showed some small differences with those of the analyses with the whole sample. Here we find slightly stronger effects for supervisor support (i.e., decrease in control group), cynicism (i.e., significant increase in control group while only marginally significant result in original analyses), and work engagement (i.e., increase in coaching group), all in the same direction of the results including the complete sample as shown by post-hoc comparisons.

Department affiliation. We compared physicians affiliated with the pediatrics department with physicians affiliated with other departments on contextual variables that could

[1] Tables summarizing the results can be requested from the first author.

For peer review only - http://bmjopen.bmj.com/site/about/guidelines.xhtml

potentially influence treatment effectiveness, that is, experienced competition and psychological safety. Experienced competition referred to the amount of competition experienced between co-workers and was measured with 5 items from Van Vianen[31]. Psychological safety referred to “a shared belief held by members of a team that the team is safe for interpersonal risk-taking”[32] allowing team members to express ideas, concerns or errors and was measured with 9 adapted items from Edmondson[32] and Van Dyck[33]. We conducted analysis of variance with competition and psychological safety measured at baseline as outcome variables. The analyses revealed that our two groups, pediatricians (n = 89) vs. ‘other’ (n = 25) did not differ with regard to both competition and psychological safety, with both p’s >.05.

Conclusion results. These analyses revealed that participants in the coaching group experienced gains, including decreased job demands, increased personal resources, and a reduction of burnout symptoms: participants perceived less job insecurity and work-family conflict, reported more autonomy and stronger personal resources, and showed a decrease in exhaustion, which is the main component of the burnout syndrome. The additional analyses conducted to test for potential effects of hospital or department affiliation on the intervention effectiveness indicated no drastic changes compared to the original analyses except that - when controlling for hospital affiliation - participants in the coaching group reported increased work engagement while no such change occurred in the control group. For all other outcome variables, neither hospital nor department affiliation influenced the effect of the intervention in a significant way, allowing us to conclude that the effect of the intervention is largely stable across the hospital organizations and department affiliations involved in this study.

DISCUSSION

Principal findings

For peer review only - http://bmjopen.bmj.com/site/about/guidelines.xhtml

Burnout rates among medical residents and specialists are on the rise[2]. Consequently, calls for action that target the professional culture and the working environment (e.g., excessive job demands) in the medical profession have been put forward [34-37]. While urgently needed, system-level changes take time. Consequently, it is imperative to develop effective measures that boost resources in order to empower physicians to effectively deal with the extreme demands they face. Although coaching is frequently advised as an intervention for physicians with burnout, surprisingly, research on its effectiveness to create personal resources and prevent burnout in the medical field barely exists [6, 38-40]. Potential remedies for physician burnout that have been put forward are mostly programs that tend to focus on curing the symptoms of burnout, rather than preventing its onset. That is, these programs focus on mindfulness, resilience or coping[41-43]. Here, we have shown that individual coaching is a promising route to both resolve and prevent burnout symptoms from residency onwards. In other words, coaching can kill two birds with one stone. Physicians in the coaching condition reported a decrease in exhaustion, the primary symptom and starting point of burnout[44]. Additionally, physicians showed increases in the personal resources psychological capital and self-compassion, both strong predictors of employee well-being and performance [45-47]. In line with the JD-R model[14], we may conclude that equipping physicians with personal resources can be a decisive factor in the prevention of burnout. That is, when physicians expand their personal resources, their ability to impact the environment increases[48], enhancing the chance that they will feel equipped to face stressful job demands and ultimately preventing burnout.

Strengths and weaknesses

To our knowledge our study provides first evidence from a controlled intervention study on the effectiveness of coaching in both medical residents and specialists. Additionally, the two-wave design including a control group together with the additional analyses we conducted

For peer review only - http://bmjopen.bmj.com/site/about/guidelines.xhtml

allow for a sound interpretation of the intervention effects. However, it should be noted that the current study is limited by its quasi-experimental design. The initial differences between the groups may be the result of appropriate self-selection or may point towards a regression to the mean effect. As such, the implications of our study should be read with care. Second, although our analyses did not suggest that hospital or department affiliation influenced treatment effectiveness greatly, the multisite character of the study including different hospital and department affiliations in the groups limits our study's potential to draw causal conclusion. Third, our study design does not allow long-term inferences of coaching effectiveness. And finally, the coaching group consisted exclusively of pediatric residents and physicians. Consequently, more research is needed that evaluates the effectiveness of coaching in different specialties, allowing broader generalization for coaching effectiveness among healthcare professionals.

Strengths and weaknesses in relation to other studies

Intervention studies in healthcare are scarce. However, a recent study investigating the effects of coaching on physician well-being and distress has found that specialists that received 3.5 hours of coaching by telephone showed a reduction in burnout symptoms and improvements in overall quality of life and resilience[6]. While this study highlights the potential of coaching for specialists, the coaching method is not comparable to face-to-face coaching which makes comparison to our study difficult. Both studies however show that coaching leads to a reduction in burnout symptoms. Importantly, our study adds evidence that coaching improves well-being and fosters personal resources among residents too. These results suggest that coaching can be beneficial to healthcare professionals from residency onwards.

Possible explanations and implications

For peer review only - http://bmjopen.bmj.com/site/about/guidelines.xhtml

Our study provides initial evidence that coaching may also function as a preventive tool through development of personal resources rather than a cure only. It also shows that only six individual coaching sessions, can reduce burnout symptoms. We therefore hope that our results inspire healthcare practitioners and policy makers to prioritize prevention rather than symptom alleviation. While collective action is sorely needed to bring changes on a system level, interventions like coaching can empower the whole spectrum of healthcare professionals from residents onwards to impact the healthcare system and eventually improve quality of care.

Unanswered questions and future research

This study shows that professional coaching can reduce burnout symptoms and strengthen personal resources. However, it is unclear how robust these effects are over time, and if effects can be generalized across different medical specialties. Additionally, the working mechanisms of coaching are yet to be discovered, making these important inquiries for the future.

For peer review only - http://bmjopen.bmj.com/site/about/guidelines.xhtml

Acknowledgments

The authors wish to thank all the medical residents and attending physicians as well as the coaches who participated in this study. The authors also want to thank Axel Willemse, Sarah van den Hee, dr. Machteld van den Heuvel, and dr. Angelique Bakker-Pieper for their contribution to the design and implementation of the study.

Contributors

All authors made substantial contributions to the conception and design, and the collection and interpretation of the data. LS, AEMvV, and JK analyzed the data and interpreted the data together with TT, APJdP and MdH. All authors reviewed and approved the final manuscript.

Funding/Support

This research received no specific grant from any funding agency in the public, commercial or not-for-profit sectors.

Competing interests

None declared.

Ethical approval

The institutional Ethic Review Board of the University of Amsterdam waived ethical approval for this study, on December 12, 2016; document 2016-WOP-7521.

Disclaimer

None reported.

Data sharing statement

For peer review only

Data and statistical code are available upon request. Any queries should be directed to the first author.

Previous presentations

This research has been presented at the following international conferences:

Coaching in Leadership and Healthcare Conference, Institute of Coaching, Boston, United States (September 29, 2018)

International Association for Medical Education AMEE Conference, Vienna, Austria (August 26, 2019)

For peer review only

References

1. Thomas NK. Resident burnout. JAMA 2004; 292:2880-2889.
2. Shanafelt TD, Hasan O, Dyrbye LN, et al. Changes in burnout and satisfaction with work-life balance in physicians and the general US working population between 2011 and 2014. Mayo Clin Proc 2015; 90:1600-1613.
3. Shanafelt TD, Sloan JA, Habermann TM. The well-being of physicians. Am J Med 2003; 114:513-519.
4. Panagioti M, Geraghty K, Johnson J, et al. Association between physician burnout and patient safety, professionalism, and patient satisfaction: a systematic review and meta-analysis. JAMA Intern Med 2018; 178:1317-1331.
5. Grant AM. The impact of life coaching on goal attainment, metacognition and mental health. Soc Behav Personal 2003; 31:253-263.
6. Dyrbye LN, Shanafelt TD, Gill PR, et al. Effect of a professional coaching intervention on the well-being and distress of physicians: A pilot randomized clinical trial. JAMA Intern Med 2019; 179:1406-1414.
7. Firth-Cozens J. Interventions to improve physicians' well-being and patient care. Soc Sci Med 2001; 52:215-222.
8. Firth-Cozens J. Doctors their wellbeing, and their stress. It's time to be proactive about stress–and to prevent it. BMJ 2003; 326:670-671.
9. Manek N. Coaching for doctors: a road to salvation. BMJ 2004; 329(7458), s29-s29.
10. Lovell B. What do we know about coaching in medical education? A literature review. Med Educ 2018; 52:376-390.
11. Theeboom T, Beersma B, van Vianen AE. Does coaching work? A meta-analysis on the effects of coaching on individual level outcomes in an organizational context. The J Pos Psychol 2014; 9:1-18.

For peer review only - http://bmjopen.bmj.com/site/about/guidelines.xhtml

12. Biswas-Diener R. Personal coaching as a positive intervention. J Clin Psychol 2009; 65:544-553.

13. Demerouti E, Bakker AB, Nachreiner F, Schaufeli WB. The job demands-resources model of burnout. J Appl Psychol 2001; 86:499-512.

14. Xanthopoulou D, Bakker AB, Demerouti E, Schaufeli WB. The role of personal resources in the job demands-resources model. Int J Stress Manag 2007; 14:121-141.

15. Spector PE, Jex SM. Development of four self-report measures of job stressors and strain: interpersonal conflict at work scale, organizational constraints scale, quantitative workload inventory, and physical symptoms inventory. J Occup Health Psychol, 1998; 3:356-367.

16. De Witte H. Arbeidsethos en jobonzekerheid: Meting en gevolgen voor welzijn, tevredenheid en inzet op het werk [Work ethics and job insecurity: Measurement and consequences for well-being, satisfaction, and work engagement]. In: Bouwen R, De Witte K, De Witte H, Taillieu T. Van groep naar gemeenschap. Liber Amicorum Prof. Dr. Leo Lagrou. Leuven, Belgium: Garant. 2000:325-350.

17. Netemeyer RG, Boles JS, McMurrian R. Development and validation of work–family conflict and family–work conflict scales. J Appl Psychol. 1996; 81:400-410.

18. Morgeson FP, Humphrey SE. The Work Design Questionnaire (WDQ): developing and validating a comprehensive measure for assessing job design and the nature of work. J Appl Psychol. 2006; 91:1321-1329.

19. Vinokur A, Schul Y, Caplan RD. Determinants of perceived social support: Interpersonal transactions, personal outlook, and transient affective states. J Pers Soc Psychol. 1987; 53:1137-1145.

20. Luthans F, Youssef CM, Avolio BJ. Psychological capital: Developing the human competitive edge. Oxford: Oxford University Press; 2007.

For peer review only - http://bmjopen.bmj.com/site/about/guidelines.xhtml

21. Schwarzer R, Jerusalem M. Generalized self-efficacy scale. In Weinman J, Wright S, Johnston M, eds. Measures in Health Psychology: A User's Portfolio. Causal and Control Beliefs. Windsor, UK: NFER-NELSON; 1995:35-37.

22. Vink J, Ouweneel E, Le Blanc P. Psychologische energiebronnen voor bevlogen werknemers: Psychologisch kapitaal in het Job Demands-Resources model. Gedrag en Organisatie. 2011; 24:101-120.

23. Neff KD, Vonk R. Self-compassion versus global self-esteem: Two different ways of relating to oneself. J Pers. 2009; 77:23-50.

24. Bond FW, Lloyd J, Guenole N. The work-related acceptance and action questionnaire: Initial psychometric findings and their implications for measuring psychological flexibility in specific contexts. J Occup Organ Psychol. 2013; 86:331-347.

25. Schaufeli WB, van Dierendonck D. UBOS Utrechtse Burnout Schaal: Handleiding. Lisse, The Netherlands: Swets Test Publishers; 2000.

26. Maslach C, Jackson SE, Leiter MP, Schaufeli WB, Schwab RL. Maslach burnout inventory. Palo Alto, CA: Consulting Psychologists Press; 1986; 21:3463-3464.

27. Schaufeli WB, Martinez IM, Pinto AM, Salanova M, Bakker AB. Burnout and engagement in university students: A cross-national study. J Cross Cult Psychol. 2002; 33:464-481.

28. Rø KEI, Gude T, Tyssen R, Aasland OG. Counselling for burnout in Norwegian doctors: one year cohort study. BMJ. 2008; 337:a2004.

29. Van Breukelen GJ. ANCOVA versus change from baseline had more power in randomized studies and more bias in nonrandomized studies. J Clin Epidemiol 2006; 59:920-925.

For peer review only - http://bmjopen.bmj.com/site/about/guidelines.xhtml

30. Huberty CJ, Morris JD. Multivariate analysis versus multiple univariate analyses. Psychol Bull 1989; 105:302-308.

31. Van Vianen, A.E.M. Person-Organization Fit: The match between newcomers' and recruiters' preferences for organizational cultures. Pers Psychol 2000; 53:113-149.

32. Edmondson A. Psychological safety and learning behavior in work teams. Adm Sci Q 1999; 44:350-383.

33. Van Dyck C, Frese M, Baer M, Sonnentag S. Organizational error management culture and its impact on performance: a two-study replication. J Appl Psychol 2005; 90:1228-1240.

34. Lemaire J, Wallace JE. Burnout among doctors. BMJ (Online) 2017; 358.

35. Wright AA, Katz IT. Beyond burnout - redesigning care to restore meaning and sanity for physicians. New Engl J Med 2018; 378:309-311.

36. Dzau VJ, Kirch DG, Nasca TJ. To care is human—collectively confronting the clinician-burnout crisis. New Engl J Med 2018; 378:312-314.

37. Eisenstein L. To fight burnout, organize. New Engl J Med 2018; 379:509-511.

38. Lovell B. What do we know about coaching in medical education? A literature review. Med Edu 2018, 52:376-390.

39. Green LS, Oades LG, Grant AM. Cognitive-behavioral, solution-focused life coaching: Enhancing goal striving, well-being, and hope. J Posit Psychol 2006; 1:142-149.

40. Grant AM, Green LS, Rynsaardt J. Developmental coaching for high school teachers: Executive coaching goes to school. Consult Psychol J: Pract Res 2010; 62:151-168.

41. Ireland MJ, Clough B, Gill K, Langan F, O'Connor A, Spencer L. A randomized controlled trial of mindfulness to reduce stress and burnout among intern medical practitioners. Med Teach 2017; 39:409-414.

For peer review only - http://bmjopen.bmj.com/site/about/guidelines.xhtml

42. Wright B, Mynett JR. Training medical students to manage difficult circumstances-a curriculum for resilience and resourcefulness? BMC Med Educ 2019; 19:280.

43. Panagioti M, Panagopoulou E, Bower P, et al. Controlled interventions to reduce burnout in physicians: a systematic review and meta-analysis. JAMA Intern Med 2017; 177:195-205.

44. Maslach C, Schaufeli WB, Leiter MP. Job burnout. Annu Rev Psychol 2001; 52:397-422.

45. Luthans F, Avolio BJ, Avey JB, Norman SM. Positive psychological capital: Measurement and relationship with performance and satisfaction. Pers Psychol 2007; 60:541-572.

46. Avey JB, Luthans F, Smith RM, Palmer NF. Impact of positive psychological capital on employee well-being over time. J Occu Health Psychol 2010; 15:17-28.

47. Neff KD. The role of self-compassion in development: A healthier way to relate to oneself. Hum Dev 2009; 52:211-214.

48. Hobfoll SE, Johnson RJ, Ennis N, Jackson AP. Resource loss, resource gain, and emotional outcomes among inner city women. J Pers Soc Psychol 2003; 84:632-643.

For peer review only

For peer review only - http://bmjopen.bmj.com/site/about/guidelines.xhtml

Figure Titles

Figure 1. Flow chart of Study Inclusion for Participants in Coaching and Control Group in a Study on Coaching Effectiveness for Medical Residents and Specialists

Figure 2. Graphic Representation of the Outcomes at Baseline and Follow-up Measurement for the Coaching Group and the Control Group in a Study on Coaching Effectiveness for Medical Residents and Specialists

For peer review only

Figure 1. Flow Chart of Study Inclusion for Participants in Coaching and Control Group in a Study on Coaching Effectiveness for Medical Residents and Specialists

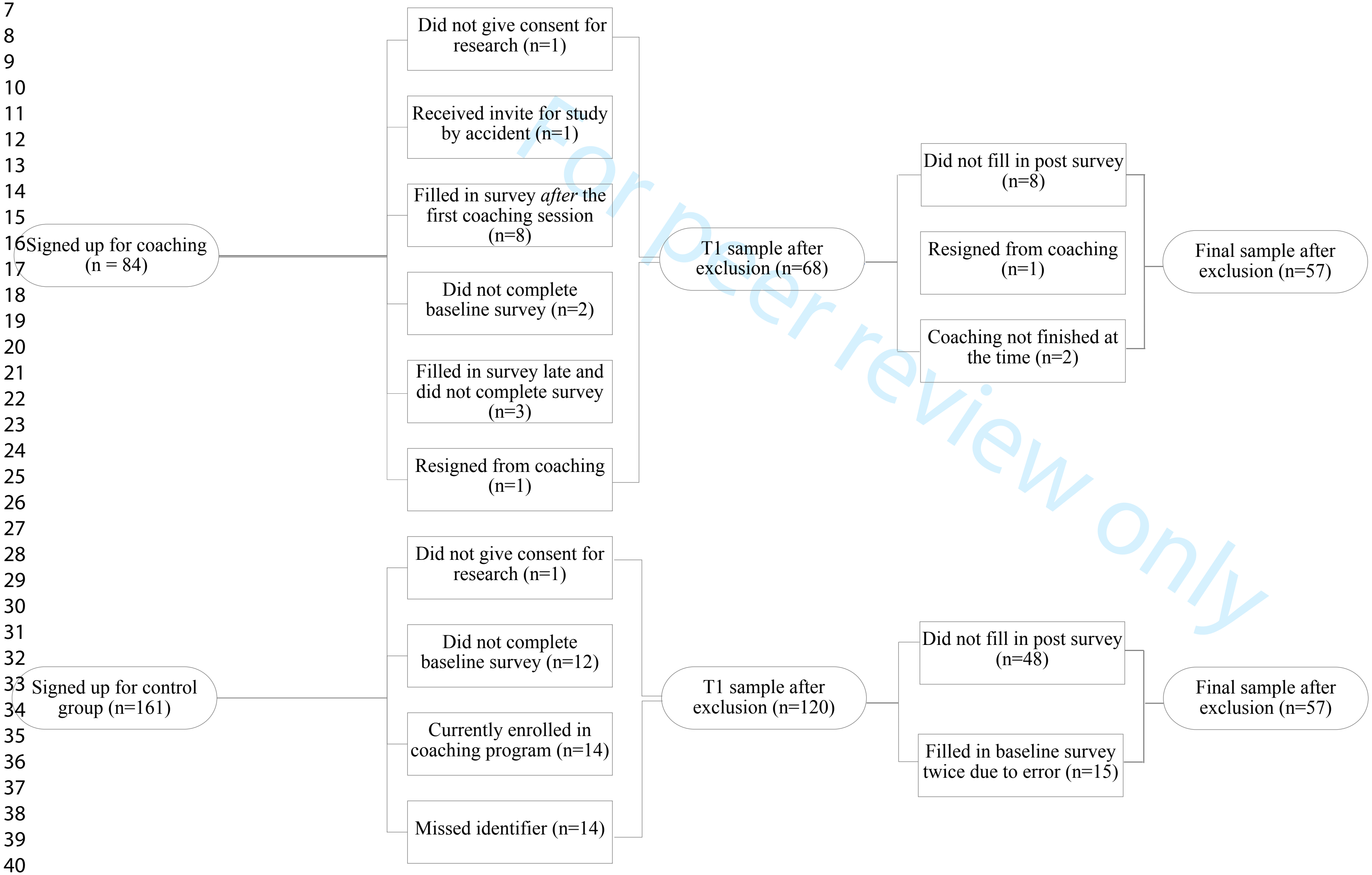

For peer review only

Figure 2. Graphic Representation of the Outcomes at Baseline and Follow-up Measurement for the Coaching Group and the Control Group in a Study on Coaching Effectiveness for Medical Residents and Specialists

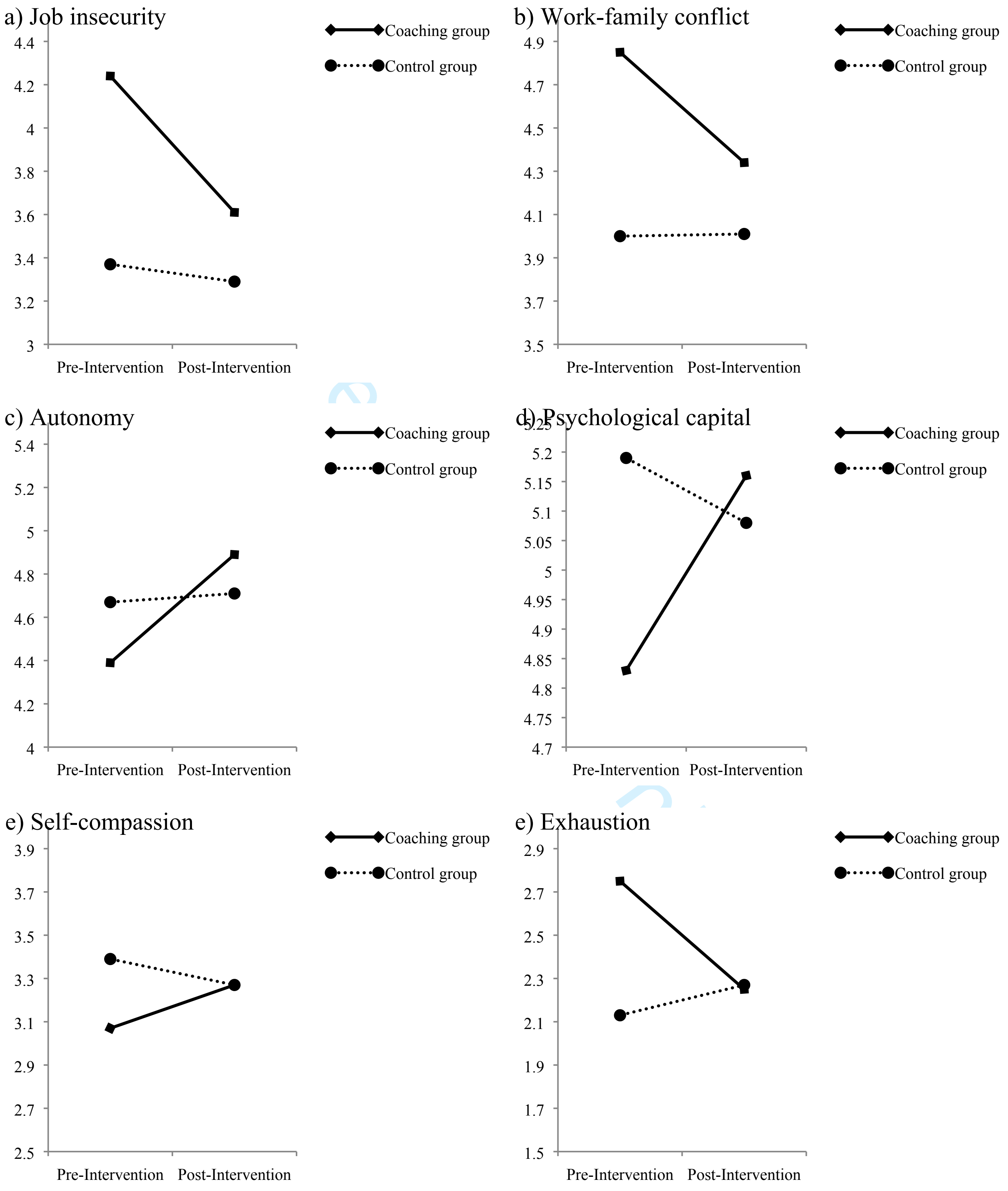

STROBE 2007 (v4) checklist of items to be included in reports of observational studies in epidemiology*
Checklist for cohort, case-control, and cross-sectional studies (combined)

Section/Topic	Item #	Recommendation	Reported on page #
**Title and abstract**	1	(*a*) Indicate the study's design with a commonly used term in the title or the abstract	1
		(*b*) Provide in the abstract an informative and balanced summary of what was done and what was found	3-4
**Introduction**			
Background/rationale	2	Explain the scientific background and rationale for the investigation being reported	6-7
Objectives	3	State specific objectives, including any pre-specified hypotheses	7
**Methods**			
Study design	4	Present key elements of study design early in the paper	7-8
Setting	5	Describe the setting, locations, and relevant dates, including periods of recruitment, exposure, follow-up, and data collection	7-8
Participants	6	(*a*) *Cohort study*—Give the eligibility criteria, and the sources and methods of selection of participants. Describe methods of follow-up<br>*Case-control study*—Give the eligibility criteria, and the sources and methods of case ascertainment and control selection. Give the rationale for the choice of cases and controls<br>*Cross-sectional study*—Give the eligibility criteria, and the sources and methods of selection of participants	7-8, 12-13 (Table 1), Figure 1
		(*b*) *Cohort study*—For matched studies, give matching criteria and number of exposed and unexposed<br>*Case-control study*—For matched studies, give matching criteria and the number of controls per case	-
Variables	7	Clearly define all outcomes, exposures, predictors, potential confounders, and effect modifiers. Give diagnostic criteria, if applicable	8-11
Data sources/ measurement	8*	For each variable of interest, give sources of data and details of methods of assessment (measurement). Describe comparability of assessment methods if there is more than one group	8-11
Bias	9	Describe any efforts to address potential sources of bias	11-12
Study size	10	Explain how the study size was arrived at	8, Figure 1
Quantitative variables	11	Explain how quantitative variables were handled in the analyses. If applicable, describe which groupings were chosen and why	11-13
Statistical methods	12	(*a*) Describe all statistical methods, including those used to control for confounding	11-14
		(*b*) Describe any methods used to examine subgroups and interactions	11-13
		(*c*) Explain how missing data were addressed	8, Figure 1
		(*d*) *Cohort study*—If applicable, explain how loss to follow-up was addressed<br>*Case-control study*—If applicable, explain how matching of cases and controls was addressed	8, Figure 1

For peer review only

		Cross-sectional study—If applicable, describe analytical methods taking account of sampling strategy	
		(*e*) Describe any sensitivity analyses	-
**Results**			
Participants	13*	(a) Report numbers of individuals at each stage of study—eg numbers potentially eligible, examined for eligibility, confirmed eligible, included in the study, completing follow-up, and analysed	14, Figure 1
		(b) Give reasons for non-participation at each stage	Figure 1
		(c) Consider use of a flow diagram	Figure 1
Descriptive data	14*	(a) Give characteristics of study participants (eg demographic, clinical, social) and information on exposures and potential confounders	12-13 (Table 1), 14
		(b) Indicate number of participants with missing data for each variable of interest	-
		(c) *Cohort study*—Summarise follow-up time (eg, average and total amount)	8
Outcome data	15*	*Cohort study*—Report numbers of outcome events or summary measures over time	15-16, Figure 2
		Case-control study—Report numbers in each exposure category, or summary measures of exposure	
		Cross-sectional study—Report numbers of outcome events or summary measures	
Main results	16	(*a*) Give unadjusted estimates and, if applicable, confounder-adjusted estimates and their precision (eg, 95% confidence interval). Make clear which confounders were adjusted for and why they were included	14-17
		(*b*) Report category boundaries when continuous variables were categorized	-
		(*c*) If relevant, consider translating estimates of relative risk into absolute risk for a meaningful time period	-
Other analyses	17	Report other analyses done—eg analyses of subgroups and interactions, and sensitivity analyses	18-19
**Discussion**			
Key results	18	Summarise key results with reference to study objectives	20
Limitations	19	Discuss limitations of the study, taking into account sources of potential bias or imprecision. Discuss both direction and magnitude of any potential bias	21-22
Interpretation	20	Give a cautious overall interpretation of results considering objectives, limitations, multiplicity of analyses, results from similar studies, and other relevant evidence	22
Generalisability	21	Discuss the generalisability (external validity) of the study results	21-22
**Other information**			
Funding	22	Give the source of funding and the role of the funders for the present study and, if applicable, for the original study on which the present article is based	23

*Give information separately for cases and controls in case-control studies and, if applicable, for exposed and unexposed groups in cohort and cross-sectional studies.

Note: An Explanation and Elaboration article discusses each checklist item and gives methodological background and published examples of transparent reporting. The STROBE checklist is best used in conjunction with this article (freely available on the Web sites of PLoS Medicine at http://www.plosmedicine.org/, Annals of Internal Medicine at http://www.annals.org/, and Epidemiology at http://www.epidem.com/). Information on the STROBE Initiative is available at www.strobe-statement.org.

For peer review only

BMJ Open

Turning the tide: a quasi-experimental study on a coaching intervention to reduce burnout symptoms and foster personal resources among medical residents and specialists in the Netherlands

Journal:	*BMJ Open*
Manuscript ID	bmjopen-2020-041708.R1
Article Type:	Original research
Date Submitted by the Author:	30-Nov-2020

Complete List of Authors:	Solms, Lara; University of Amsterdam, Work and Organizational Psychology; Erasmus Medical Center - Sophia Children's Hospital, Pediatrics<br>van Vianen, Annelies; University of Amsterdam, Work and Organizational Psychology<br>Koen, Jessie; University of Amsterdam, Work and Organizational Psychology<br>Theeboom, Tim; Vrije Universiteit Amsterdam, School of Business and Economics<br>de Pagter, Anne; Erasmus Medical Center - Sophia Children's Hospital , Pediatric Hematology; Leiden University Medical Center, Pediatrics<br>De Hoog, Matthijs; Erasmus Medical Center - Sophia Children's Hospital, Pediatrics/ Pediatric Intensive Care Unit

**Primary Subject Heading**:	Mental health
Secondary Subject Heading:	Medical education and training
Keywords:	EDUCATION & TRAINING (see Medical Education & Training), Human resource management < HEALTH SERVICES ADMINISTRATION & MANAGEMENT, MENTAL HEALTH

SCHOLARONE™ Manuscripts

I, the Submitting Author has the right to grant and does grant on behalf of all authors of the Work (as defined in the below author licence), an exclusive licence and/or a non-exclusive licence for contributions from authors who are: i) UK Crown employees; ii) where BMJ has agreed a CC-BY licence shall apply, and/or iii) in accordance with the terms applicable for US Federal Government officers or employees acting as part of their official duties; on a worldwide, perpetual, irrevocable, royalty-free basis to BMJ Publishing Group Ltd ("BMJ") its licensees and where the relevant Journal is co-owned by BMJ to the co-owners of the Journal, to publish the Work in this journal and any other BMJ products and to exploit all rights, as set out in our licence.

The Submitting Author accepts and understands that any supply made under these terms is made by BMJ to the Submitting Author unless you are acting as an employee on behalf of your employer or a postgraduate student of an affiliated institution which is paying any applicable article publishing charge ("APC") for Open Access articles. Where the Submitting Author wishes to make the Work available on an Open Access basis (and intends to pay the relevant APC), the terms of reuse of such Open Access shall be governed by a Creative Commons licence – details of these licences and which Creative Commons licence will apply to this Work are set out in our licence referred to above.

Other than as permitted in any relevant BMJ Author's Self Archiving Policies, I confirm this Work has not been accepted for publication elsewhere, is not being considered for publication elsewhere and does not duplicate material already published. I confirm all authors consent to publication of this Work and authorise the granting of this licence.

For peer review only

Turning the tide: a quasi-experimental study on a coaching intervention to reduce burnout symptoms and foster personal resources among medical residents and specialists in the Netherlands

Lara Solms, MSc, Annelies E.M. van Vianen, PhD, Jessie Koen, PhD, Tim Theeboom, PhD, Anne P.J. de Pagter*, MD, PhD, Matthijs de Hoog*, MD, PhD on behalf of the Challenge & Support Research Network

L. Solms is PhD candidate, Department of Work and Organizational Psychology, University of Amsterdam, Amsterdam, The Netherlands, PhD candidate, Department of Pediatrics, Erasmus MC-Sophia Children's Hospital, Rotterdam, The Netherlands

A.E.M van Vianen is professor of work and organizational psychology, Department of Work and Organizational Psychology, University of Amsterdam, Amsterdam, The Netherlands

T. Theeboom is assistant professor, School of Business and Economics, Vrije Universiteit Amsterdam, Amsterdam, The Netherlands

J. Koen is assistant professor, Department of Work and Organizational Psychology, University of Amsterdam, Amsterdam, The Netherlands

A.P.J. de Pagter is a pediatrician-hematologist, Department of Pediatrics, Erasmus MC-Sophia Children's Hospital, Rotterdam, The Netherlands

M. de Hoog is pediatric intensivist, professor of postgraduate medical education, director of pediatric residency training & director of PICU, Erasmus MC-Sophia Children's Hospital, Rotterdam, The Netherlands

The Challenge & Support Research Network:

Annemarie M.C. van Rossum, Wouter J.W. Kollen, Robbert G.M. Bredius, Amando J. Heesterman, Marlies A. van Houten, Marie-José E. Walenkamp, Adrienne A.M. Zandbergen, Stephanie C.E. Schuit, Jacoline E.C. Bromberg, Axel Willemse, Sarah M. van den Hee, Machteld van den Heuvel, and Angelique Bakker-Pieper.

For peer review only - http://bmjopen.bmj.com/site/about/guidelines.xhtml

*A.P.J. de Pagter and M. de Hoog contributed equally to this paper.

Correspondence should be addressed to Anne P.J. de Pagter, Department of Pediatrics, Erasmus MC-Sophia Children's Hospital, Rotterdam, Sp-2430. Wytemaweg 80, 3015 CN Rotterdam, The Netherlands; telephone: +31107032435; e-mail: p.depagter@erasmusmc.nl

Manuscript word count: 4113

Keywords: Coaching; professional development; burnout; personal resources; residents; specialists

For peer review only

For peer review only - http://bmjopen.bmj.com/site/about/guidelines.xhtml

ABSTRACT

Objectives

Physician burnout is increasing, starting already among residents. The consequences of burnout are not limited to physicians' well-being, they also pose a threat to patient care and safety. This study investigated the effectiveness of a professional coaching intervention to reduce burnout symptoms and foster personal resources in residents and specialists.

Design

In a controlled field experiment, medical residents and specialists received six coaching sessions, while a control group did not undergo any treatment. The authors assessed burnout symptoms of exhaustion and cynicism, the personal resources psychological capital, psychological flexibility, and self-compassion, as well as job demands and job resources with validated questionnaires (January 2017 until August 2018). The authors conducted repeated measures analyses of variance (ANOVA) procedures to examine changes over time for the intervention and the control group.

Setting

Four academic hospitals in the Netherlands.

Participants

A final sample of 57 residents and specialists volunteered in an individual coaching program. A control group of 57 physicians did not undergo any treatment.

Intervention

Coaching was provided by professional coaches during a period of approximately 10 months aiming at personal development and growth.

For peer review only - http://bmjopen.bmj.com/site/about/guidelines.xhtml

Results

The coaching group (response rate 68%, 57 physicians, 47 women) reported a reduction in burnout symptoms and an increase in personal resources after the coaching intervention, while no such changes occurred in the control group (response rate 35%, 42 women), as indicated by significant Time x Group interactions, all *p*s < .01. Specifically, physicians increased their psychological capital (η_p^2 = .139), their self-compassion (η_p^2 = .083), and reported significantly less exhaustion (η_p^2 = .126), the main component of the burnout syndrome.

Conclusion

This study suggests that individual coaching is a promising route to reduce burnout symptoms in both residents and specialists. Moreover, it strengthens personal resources that play a crucial role in the prevention of burnout.

Article summary

Strengths and limitations of this study

- This study provides first evidence from a controlled intervention study on the effectiveness of coaching in both medical residents and specialists.
- Six individual professional face-to-face coaching sessions can decrease burnout symptoms (i.e., exhaustion) among medical residents and specialists.
- Preventive coaching contributes to the improvement of the personal resources psychological capital and self-compassion, resources that play a role in the prevention of burnout.
- The study is limited by its quasi-experimental design. However, the analyses controlled for initial differences between the coaching and the control group.

For peer review only

- The coaching group consisted exclusively of pediatric residents and physicians. Consequently, more research is needed that evaluates the effectiveness of coaching in different specialties, allowing broader generalization for coaching effectiveness among healthcare professionals.

For peer review only

For peer review only - http://bmjopen.bmj.com/site/about/guidelines.xhtml

INTRODUCTION

Physicians experience a variety of stressors including time pressure, emotionally taxing patient-interactions, and an increasing bureaucratic burden. Not surprisingly, burnout (i.e., feeling exhausted, dissociated and less efficient) is high among senior healthcare professionals as well as residents.[1-2] Burnout has severe consequences for physicians, often leading to long-term absenteeism and eventually abandonment of the medical profession.[3] But the negative consequences are not limited to physicians' well-being and careers: with burnout flooding the healthcare system, patient safety is also at risk. Physician burnout is associated with poorer quality of care and reduced patient safety.[4-5]

In order to reduce the risk of physician burnout, and thus warrant adequate patient care and patient safety, powerful interventions are needed that prioritize physicians' needs . This is the case in professional coaching, which is commonly defined as "a result-oriented, systematic process in which the coach facilitates the enhancement of life experience and goal-attainment in the personal and/or professional life of normal, non-clinical clients."[6] This definition of coaching acts on the assumption that coaching is a facilitative process aimed a self-directed change of the client.[7] Additionally, this definition distinguishes coaching from other helping relationships such as mentoring and counseling.[8] Mentoring generally refers to a relationship between a more senior employee and a protégé aimed at offering guidance and feedback in a specific organizational context.[9] In coaching, a coach usually does not hold a formal position within the client's organization. Additionally, our definition of coaching emphasizes a non-clinical target group, which makes it clearly distinguishable from counseling and therapy.

Surprisingly, coaching is not common in medical practice and research is scarce[10-14] despite the fact that the positive effects of coaching on well-being and functioning have been

For peer review only - http://bmjopen.bmj.com/site/about/guidelines.xhtml

demonstrated in a number of educational and professional settings.[15] Furthermore, with coaching being generally connected to problem elimination (e.g., burnout) in healthcare, rather than to professional development and well-being, its power is underrated if not invisible due to stigma. Given the potential benefits of coaching for physician well-being, research on the effectiveness of coaching in a professional development setting is sorely needed.

A professional coaching intervention may simultaneously help to resolve and prevent burnout among physicians. That is, professional coaching can not only directly reduce burnout symptoms, but can also strengthen personal resources that may prevent such burnout symptoms in the first place.[16] This assumption is rooted in research on burnout, which shows that the onset of burnout is caused by both heavy job demands and a lack of (personal) resources.[17] Personal resources refer to "aspects of the self that are generally linked to resiliency and refer to individuals' sense of their ability to control and impact upon their environment successfully."[18] According to the Job Demands-Resources Model (JD-R),[17] a common work-stress model in the prediction of burnout and work engagement, personal resources help people to deal with extreme demands, ultimately buffering the negative effects of job demands on burnout.[19] At the same time, personal resources stimulate motivation and work engagement. With both work engagement and well-being (i.e., a lack of burnout) being indispensable for optimal physician functioning, the value of professional coaching lies in its ability to kill two birds with one stone: It aims to reduce burnout symptoms as well as stimulate life-long reflection and self-management through recognizing and strengthening individuals' personal resources.

In this two-wave quasi-experimental study we evaluated the benefits of an individual coaching program for the resources, demands, and well-being (i.e., lack of burnout symptoms), and work engagement of medical residents and specialists in the Netherlands.

For peer review only - http://bmjopen.bmj.com/site/about/guidelines.xhtml

METHOD

Study Setting and Population

This study evaluates the effectiveness of an individual coaching program in two major academic hospitals, the Erasmus Medical Center (EMC) and the Leiden University Medical Center (LUMC) in the Netherlands. Using an quasi-experimental pre-test post-test control design, this study comprises the comparison of a treatment group (i.e., coaching group) with a control group that did not receive any treatment on two measurement occasions (i.e., at pre- and post-test). In a quasi-experimental design like this, the assignment to conditions (i.e., coaching versus no coaching) is non-random.[20] A final number of 114 physicians participated in this study of which 57 received individual coaching between January 2017 and August 2018. The coaching program was completely voluntary, offering six individual coaching sessions to both residents and specialists from the pediatrics department at the EMC and LUMC. Because funding for the coaching program was initially only available for the pediatrics department, physicians from other departments (i.e., internal medicine, neurology) and pediatric residents from two other hospitals served as a control group. Additionally, pediatricians who did not voice interest in the coaching program were placed in the control group as well. See Table 1 for sample characteristics. The study protocol was approved by the institutional Ethics Review Board of the University of Amsterdam.

For peer review only

Intervention and Procedure

Physicians were informed through different channels (i.e., e-mail newsletter, information presentation, mouth-to-mouth) about the coaching program and could sign themselves up for the program via e-mail. Physicians that voiced interest in the coaching program, were asked to participate in the study and were able to choose a coach of their preference. All coaches participating in the program were selected based on a number of relevant criteria such as years of experience and affinity and experience with the medical profession. Specifically, all

For peer review only - http://bmjopen.bmj.com/site/about/guidelines.xhtml

coaches were selected based on their senior level of coaching experience, their experience with physician-clients, positive references from previous physician clients, and accredited coaching training. The selection committee consisted of a coaching professional, a senior human resources manager, and the medical specialist and initiator of the coaching program. Physicians could view introductory videoclips of coaches on the program website. In these 1-minute long videos, coaches introduced themselves and provided information about their way of working with clients. Thereafter, physicians chose their coach and the first coaching session was arranged.

The coaching process. Coaches and participants received ample freedom to shape the coaching program according to coaches' professional methods and participants' needs. Because an important premise of successful coaching is that the coach and the client agree on the goals to achieve, as well as the means to achieve them,[21-22] we largely avoided regulations to the coaching process (such as the topics of the coaching, the coaching method or the speed of the trajectories) that might have stood in the way of such consensus. Constraints were set only with regard to the overall outline of the coaching program. That is, coaching was set to a maximum of 6 (1 or 1.5 hour long) sessions and coaches and participants were encouraged to complete the coaching trajectories within a period of approximately 10 months but could stretch their trajectories if necessary (M = 7.98, SD = 2.81), which only few participants did. All participants started their coaching trajectory individually depending on the availability of their coach. Time in between coaching sessions was determined by the participants – and hence varied – and was further not registered. All coaching sessions took place face-to-face and outside of work at the coach's workspace. Informed consent was obtained from all participants in both the coaching and the control group at the beginning of the study. Participants who did not give consent, were excluded from the study. Demographics as well as the study variables were measured with an online survey delivered via Qualtrics (Qualtrics,

For peer review only - http://bmjopen.bmj.com/site/about/guidelines.xhtml

2005) shortly before the first coaching session at baseline (T1) and minimal 7 days (M = 87.25, SD = 92.95, range: 7-364) after the last coaching session was finished (T2). Participants that failed to fill out the T1 or T2 survey at first, received up to three reminders by e-mail with the request to complete the survey. For a description of exclusion criteria, see Figure 1.

Study Variables

In line with the JD-R model, we measured job demands (workload, job insecurity, work-family conflict), job resources (autonomy, colleague support, supervisor support), personal resources (psychological capital, self-compassion, psychological flexibility), as well as burnout symptoms and work engagement.

Job demands. We measured workload, job insecurity, and work-family conflict.

Workload was assessed with four items from the Quantitative Workload Inventory[23] and two additional items that were added to match the specific demands of medical practice. The two additional items assessed working overtime and emotional strain. All items were measured on a 5-point scale ranging from 1 (“never”) to 5 (“always”).

Job insecurity, that is, “the perceived threat of job loss and the worries related to that threat” was assessed with a 5-item adapted version of the Job Insecurity Scale.[24] The items were scored on a 7-point scale ranging from 1 (“not at all applicable”) to 7 (“very applicable”).

Work-Family Conflict was measured with four items of the Work-Family Conflict Scale[25] assessing “the general demands of, time devoted to, and strain created by the work interfere with performing family-related responsibilities.” The items were scored on a 7-point scale ranging from 1 (“not at all applicable”) to 7 (“very applicable”).

Job resources. Job resources encompassed autonomy, supervisor support, and colleague support.

For peer review only - http://bmjopen.bmj.com/site/about/guidelines.xhtml

Autonomy was measured with nine items from the Work Design Questionnaire[26] assessing perceived autonomy with regard to work scheduling and methods, and decision-making. The items were scored on a 7-point scale ranging from 1 (“totally disagree”) to 7 (“totally agree”).

Supervisor support, that is, the experienced psychological and work support from the supervisor was assessed with six items from Vinokur, Schul, and Caplan.[27] For residents, supervisory support measured the support received from the training supervisor, whereas for specialists, supervisory support measured the support received from the head of the department. The items were scored on a 7-point scale ranging from 1 (“totally disagree”) to 7 (“totally agree”).

Colleague support, the experienced psychological and work support from colleagues, was assessed with the same six items as supervisor support,[27] but the items referred to colleagues instead of the supervisor.

Personal resources. We measured psychological capital, self-compassion, and psychological flexibility.

To capture *Psychological capital*’s components, hope, optimism, and resilience, we used 9 items from the Dutch version of the PsyCap questionnaire.[28] To measure the fourth component, self-efficacy, we used three items based on the Generalized Self-efficacy Scale[29] that were adapted so they would fit the occupational setting as used in previous research.[30] The items were scored on a 7-point scale ranging from 1 (“totally disagree”) to 7 (“totally agree”).

Self-compassion, that is “treating oneself with kindness, recognizing one’s shared humanity, and being mindful when considering negative aspects of oneself” was measured with six items from the Self-Compassion Scale.[31] This scale encompasses three subscales: self-kindness, common humanity and mindfulness. The items were scored on a 5-point scale ranging from 1 (“rarely”) to 5 (“almost always”).

For peer review only - http://bmjopen.bmj.com/site/about/guidelines.xhtml

Psychological flexibility, that is, the ability to flexibly take appropriate action towards achieving goals and values, even in the presence of challenging or unwanted events was measured with seven items of the Work Acceptance and Action Questionnaire.[32] The items were scored on a 5-point scale ranging from 1 (“rarely”) to 5 (“almost always”).

Burnout symptoms and work engagement.

Burnout symptoms. We measured burnout symptoms with the two core scales exhaustion and cynicism of the Dutch version of the Maslach Burnout Inventory.[33-34] Both scales were measured with five and four items respectively. The items were scored on a 7-point scale ranging from 1 (“totally disagree”) to 7 (“totally agree”).

Work engagement. We measured work engagement with the Utrecht Work Engagement Scale.[35] Its nine items cover the three subscales vigor, dedication, and absorption. The items were scored on a 7-point scale ranging from 1 (“never”) to 7 (“always”).

Statistical Analyses

Intervention effects. To test if the coaching intervention would have beneficial effects, repeated measures analyses of variance (ANOVA) procedures were performed to examine changes over time for the intervention and the control group. The outcomes analyzed were job demands (workload, job insecurity, work-family conflict), job resources (autonomy, colleague support, supervisor support), personal resources (psychological capital self-compassion, psychological flexibility), as well as burnout symptoms (exhaustion, cynicism) and work engagement. We controlled for coaching attitude, i.e., the degree to which one believes coaching is beneficial or helpful, which was measured at baseline, because it can be expected that a positive attitude may contribute to the success of the intervention. Significant Time x Group interactions of the outcome variables were followed up with post hoc tests.

Preliminary analyses. ***Self-selection of participants***. Because participation in the coaching program was voluntarily – and complete randomization of participants to conditions was not

For peer review only - http://bmjopen.bmj.com/site/about/guidelines.xhtml

possible due to internal (i.e., financial and time) restrictions and prior agreements within the hospital organizations – we examined structural demographic differences prior to the intervention between the coaching and the control group (T1). These demographics were gender, age, tenure (i.e., medical resident, specialist), department (i.e., pediatrics, internal medicine, neurology) and hospital affiliation (i.e., EMC, LUMC, VUMC, AMC). Sample characteristics are displayed in Table 1. While both groups did not differ with respect to gender ($x^2(1) = 1.28$, $p = .26$), age ($F(1,112) = 0.49$, $p = .49$), and tenure ($x^2(1) = 0.33$, $p = .57$), they did differ in department affiliation ($x^2(2) = 32.02$, $p < .001$) and hospital affiliation ($x^2(3) = 22.55$, $p < .001$). More specifically, all coaching participants were affiliated with the pediatrics department of two of the four participating hospitals. We conducted three types of additional analyses to rule out that potential effects attributed to the coaching intervention were caused by factors related to the imbalance of department and hospital affiliation – although conceptually, this is highly unlikely. *Hospital affiliation.* To estimate a potential impact of hospital affiliation on treatment effectiveness, we conducted multiple univariate repeated measures for each of the outcome variables including hospital affiliation as additional control variable to see if the previous results would hold. Additionally, we conducted the original analyses solely for physicians employed at the two medical hospitals that were represented in the intervention group. *Department affiliation.* Given that all participants in the coaching intervention were affiliated with the pediatrics department we analyzed whether pediatricians differed from physicians affiliated with other departments (e.g., neurology, internal medicine) with respect to contextual variables, here *competition*, and *psychological safety,* variables that reflect experienced department work climate and potentially could influence treatment effectiveness.

Table 1. Characteristics of the Study Population in a Study on Coaching Effectiveness for Medical Residents and Specialists, 2017-2018[a]

	Intervention	Control

For peer review only - http://bmjopen.bmj.com/site/about/guidelines.xhtml

Characteristics	(N = 57)	(N = 57)
Male sex – no. (%)	10 (17.5)	15 (26.3)
**Age - yr**		
Median	33	35
Interquartile range	9.5	12
**Specialty – no (%)**		
Pediatrics	57 (100)	32 (56.1)
Internal medicine	-	15 (26.3)
Neurology	-	10 (17.5)
**Professional role – no (%)**		
Resident	33 (57.9)	36 (63.2)
Specialist	24 (42.1)	21 (36.8)
**Hospital – no (%)**		
EMC	32 (56.1)	33 (57.9)
LUMC	25 (43.9)	9 (15.8)
VUMC	-	7 (12.3)
AMC	-	8 (14.0)
Coaching experience – no. (%)	22 (38.6)	19 (33.3)
**Home situation – no (%)**		
Children, one or more	28 (49.1)	29 (50.9)
No children	29 (50.9)	28 (49.1)

[a]This study was conducted at 4 academic hospitals in the Netherlands. In this study, the authors investigated the effects of an individual coaching intervention on burnout symptoms, work engagement, personal resources, job demands and job resources among pediatric residents and specialists.

Baseline differences between groups. With respect to the outcome variables at baseline, we found significant differences between the intervention and the control group: The intervention group scored significantly lower on personal resources, and significantly higher on job demands and exhaustion, similar to the results of a previous study on counseling in Norwegian doctors.[36] An overview of the differences between the groups is displayed in Table 2. Because distribution of participants was not random, and because there were significant differences on a number of outcomes prior to the intervention, we tested our

For peer review only - http://bmjopen.bmj.com/site/about/guidelines.xhtml

hypotheses with repeated measures analysis of variance. These analyses are favored over the analysis of covariance in a non-randomized intervention study.[37] Additionally, we followed the recommendations of Huberty and Moris[38] and conducted multiple ANOVAs as opposed to a MANOVA as a preliminary step to multiple ANOVAs.

Table 2. Means and Standard Deviations (*SD*) of Study Variables for the Control and the Intervention Group at Baseline (T1) in a Study on Coaching Effectiveness for Medical Residents and Specialists, 2017-2018[a]

Study Variables	Intervention (N = 57)	Control (N = 57)
	Mean (*SD*)	Mean (*SD*)
**Job demands**		
Workload**	3.48 (.67)	3.10 (.78)
Job insecurity**	4.24 (1.33)	3.37 (1.45)
Work-family conflict**	4.85 (1.05)	4.00 (1.19)
**Job resources**		
Autonomy	4.39 (1.03)	4.67 (1.14)
Colleague support	5.33 (.96)	5.47 (.90)
Supervisor support	4.63 (1.51)	4.98 (1.42)
**Personal resources**		
PsyCap**	4.83 (.69)	5.19 (.72)
Self-compassion**	3.07 (.60)	3.39 (.66)
Psych. flexibility*	3.43 (.63)	3.67 (.53)
**Outcomes**		
Exhaustion**	2.75 (1.08)	2.13 (.92)
Cynicism	2.11 (1.08)	2.06 (.93)
Work engagement	5.08 (.78)	5.04 (.75)

Abbreviation: SD indicates standard deviation; PsyCap indicates psychological capital; psych. flexibility indicates psychological flexibility.
[a]Differences in means between the intervention and the control group are indicated by the following significance values: $^{\dagger}p < .10$; $^{*}p < .05$; $^{**}p < .01$.

Patient and public involvement

This study investigated the effectiveness of a professional coaching intervention in medical residents and specialists. No patients or public representatives were involved in the study.

For peer review only - http://bmjopen.bmj.com/site/about/guidelines.xhtml

RESULTS

A total number of 84 physicians signed up for the coaching program while 161 physicians signed up for the control group. Of these two groups, 57 physicians in each group completed the follow-up measurement and were included in the final sample (Figure 1). Table 1 shows the demographic characteristics of the study population. Internal consistencies ranged from .75 to .95 and were acceptable for all scales. See Table 3 for correlations between the study variables at baseline.

Intervention effects. The analyses revealed significant changes in the intervention group that did not occur in the control group, as indicated by significant Group x Time interactions for a number of outcomes. A summary of the results is shown in Table 4. With regard to job demands, post hoc analyses revealed a decrease in job insecurity and work-family conflict in the intervention group with both *p*s $< .05$. With regard to job resources, post-hoc analyses showed that autonomy increased in the intervention group, while supervisor support decreased in the control group, all *p*s $< .05$. With regard to personal resources, post hoc comparisons indicated an increase in psychological capital and self-compassion in the intervention group, all *p*s $< .05$, as well as a decrease in self-compassion in the control group, $p < .05$. No changes occurred in psychological flexibility, in either the control or coaching group, all *p*s $>.05$. Finally, with regard to outcomes, analyses showed that the coaching group significantly decreased their burnout symptoms but showed no changes in work engagement. Post hoc comparisons indicated a decrease in exhaustion in the intervention group, $p < .05$, while no such changes occurred in the control group, all *p*s $> .05$ or with regard to cynicism, $p > .05$. For a graphical representation of these effects, see Figure 2.

For peer review only - http://bmjopen.bmj.com/site/about/guidelines.xhtml

Table 3. Correlations between the Study Variables for the Intervention and Control Group* at Baseline in a Study on Coaching Effectiveness for Medical Residents and Specialists, 2017-2018[a]

Study Variables	1	2	3	4	5	6	7	8	9	10	11	12	13
1. Coaching attitude	1	.284*	-.032	.015	-.024	-.088	-.094	-.218	-.203	.172	-.014	.006	.092
2. Workload	-.050	1	-.089	.322*	.015	-.124	-.209	-.017	-.067	.12	.299*	.168	-.066
3. Job insecurity	.128	.191	1	-.066	-.458**	-.278*	.095	-.511**	-.405**	-.220	.225	.221	-.285*
4. Work-family conflict	-.142	.454**	-.071	1	.038	-.045	.131	.060	-.107	-.004	.341**	.207	-.100
5. Autonomy	-.025	-.333	-.335	-.232	1	.161	-.036	.382**	.224	.142	-.177	-.141	.290*
6. Colleague support	.234	-.163	-.153	-.040	.348	1	-.100	.418**	.348**	.137	-.463**	-.510**	.557**
7. Supervisor support	-.026	.020	-.379	-.035	.341*	.233	1	.154	-.031	-.076	.140	-.032	.027
8. PsyCap	-.173	-.370	-.463	-.199	.486**	.325*	.401**	1	.607**	.242	-.365**	-.576**	.627**
9. Self-compassion	-.176	-.276	-.422	-.260	.244	.362**	.427**	.512**	1	.086	-.545**	-.397**	.388**
10. Psych. flexibility	-.238	.031	-.170	.189	.415**	.256	.212	.273*	.209	1	-.094	-.187	.407**
11. Exhaustion	.064	.493**	.313*	.411**	-.299*	-.222	-.318*	-.363**	-.439**	-.157	1	.602**	-.570**
12. Cynicism	-.111	.355**	.356**	.005	-.339**	-.328*	-.373**	-473**	-.286*	-.337	.686**	1	-.617**
13. Work engagement	.228	-.164	-.228	-.041	.309*	.349**	.491**	.451**	.303*	.403**	-.482**	-.712	1

Abbreviation: PsyCap indicates psychological capital; psych. flexibility indicates psychological flexibility.
[a]Above the diagonal: coaching group; below the diagonal: control group

For peer review only - http://bmjopen.bmj.com/site/about/guidelines.xhtml

Table 4. Summary of Results for Repeated Measures Analyses and Pre- and Postintervention Means for the Intervention Group in a Study on Coaching Effectiveness for Medical Residents and Specialists, 2017-2018[a-c]

Time x Group interaction for study variables	Mean Square	*F*	*p*	η_p^2	Preintervention Mean (*SD*)	Postintervention Mean (*SD*)	*Df*	*t*	*p*
**Job demands**									
Workload	.211	.837	.362	.007	3.48 (.67)	3.31 (.61)	56	1.97	.053
Job insecurity**	6.07	10.99	.001	.090	4.24 (1.33)	3.61 (1.46)	56	4.10	.000
Work- family-conflict**	4.60	8.33	.005	.070	4.85 (1.05)	4.34 (1.12)	56	4.36	.000
**Job resources**									
Autonomy**	3.41	7.56	.007	.064	4.39 (1.03)	4.89 (1.06)	56	-4.19	.000
Colleague support*	1.68	4.68	.033	.040	5.33 (.96)	5.56 (.79)	56	-1.94	.057
Supervisor support*	3.79	5.60	.020	.048	4.63 (1.51)	4.82 (1.35)	56	-1.28	.207
**Personal resources**									
PsyCap**	2.57	17.92	.000	.139	4.83 (.69)	5.16 (.65)	56	-4.08	.000
Self-compassion**	1.26	10.00	.002	.083	3.07 (.60)	3.27 (.52)	56	-2.72	.009
Psych. flexibility	.335	1.80	.182	.016	3.43 (.63)	3.47 (.65)	56	-0.53	.600
**Outcomes**									
Exhaustion**	6.20	15.94	.000	.126	2.75 (1.08)	2.25 (.79)	56	4.00	.000
Cynicism*	2.52	5.44	.022	.047	2.11 (1.08)	1.90 (.75)	56	1.46	.151
Work engagement†	.69	3.19	.077	.028	5.08 (.78)	5.28 (.59)	56	-2.19	.033

[a]The following significance values are used: †$p < .10$; *$p < .05$; **$p < .01$.
[b]η_p^2 refers to the degree to which variability among observations can be attributed to conditions controlling for the subjects' effect that is unaccounted for by the model.
[c]*Df* for the time x group interaction = 1 for all study variables and 111 for the error(time)

For peer review only

Supplementary analyses

In order to rule out that effects attributed to the intervention were (partly) influenced by hospital and department affiliation we conducted three additional analyses[1].

Hospital affiliation. First, we conducted repeated measures analyses for each outcome variable with the whole sample, but this time added hospital affiliation as a control variable. The results of these analyses revealed no significant differences with those of the original analyses, except for work engagement as outcome. Here, we find (instead of a marginal significant) a significant group x time interaction, $p = .019$. Post hoc analyses indicated that the coaching group reported increased work engagement after the coaching program, with no changes occurring in the control group. Overall, these results indicate that hospital affiliation did not influence treatment effectiveness in significant ways. Additionally, we examined whether the results of the whole sample (including four hospitals) were comparable to those of a subsample including only physicians from the two academic hospitals that offered the coaching intervention (i.e., EMC and LUMC). We conducted repeated measures analyses for each outcome variable. Coaching attitude, i.e., the degree to which one believes coaching is beneficial or helpful, was included in the analyses as control variable. The results of the analyses with the subsample showed some small differences with those of the analyses with the whole sample. Here we find slightly stronger effects for supervisor support (i.e., decrease in control group), cynicism (i.e., significant increase in control group while only marginally significant result in original analyses), and work engagement (i.e., increase in coaching group), all in the same direction of the results including the complete sample as shown by post-hoc comparisons.

[1] Tables summarizing the results can be requested from the first author.

For peer review only - http://bmjopen.bmj.com/site/about/guidelines.xhtml

Department affiliation. We compared physicians affiliated with the pediatrics department with physicians affiliated with other departments on contextual variables that could potentially influence treatment effectiveness, that is, experienced competition and psychological safety. Experienced competition referred to the amount of competition experienced between co-workers and was measured with five items from Van Vianen.[39] Psychological safety referred to “a shared belief held by members of a team that the team is safe for interpersonal risk-taking”[32] allowing team members to express ideas, concerns or errors and was measured with nine adapted items from Edmondson[40] and Van Dyck[41]. We conducted analysis of variance with competition and psychological safety measured at baseline as outcome variables. The analyses revealed that our two groups, pediatricians (n = 89) versus ‘other’ (n = 25) did not differ with regard to both competition and psychological safety, with both *p*s >.05.

Conclusion results. These analyses revealed that participants in the coaching group experienced gains, including decreased job demands, increased personal resources, and a reduction of burnout symptoms: participants perceived less job insecurity and work-family conflict, reported more autonomy and stronger personal resources, and showed a decrease in exhaustion, which is the main component of the burnout syndrome. The additional analyses conducted to test for potential effects of hospital or department affiliation on the intervention effectiveness indicated no drastic changes compared to the original analyses except that – when controlling for hospital affiliation – participants in the coaching group reported increased work engagement while no such change occurred in the control group. For all other outcome variables, neither hospital nor department affiliation influenced the effect of the intervention in a significant way, allowing us to conclude that the effect of the intervention is largely stable across the hospital organizations and department affiliations involved in this study.

For peer review only - http://bmjopen.bmj.com/site/about/guidelines.xhtml

DISCUSSION

Principal findings

Burnout rates among medical residents and specialists are on the rise.[2] Consequently, calls for action that target the professional culture and the working environment (e.g., excessive job demands) in the medical profession have been put forward.[42-45] While urgently needed, system-level changes take time. Therefore, it is imperative to develop effective measures that boost resources in order to empower physicians to effectively deal with the extreme demands they face. Although coaching is frequently advised as an intervention for physicians with burnout, surprisingly, research on its effectiveness to create personal resources and prevent burnout in the medical field barely exists.[10, 46-48] Potential remedies for physician burnout that have been put forward tend to be programs that focus on curing the symptoms of burnout, rather than preventing its onset. That is, these programs focus on mindfulness, resilience or coping.[49-51] Here, we have shown that individual coaching is a promising route to both resolve and prevent burnout symptoms from residency onwards. In other words, coaching can kill two birds with one stone. Physicians in the coaching group reported a decrease in exhaustion, the primary symptom and starting point of burnout.[52] Additionally, physicians showed increases in the personal resources psychological capital and self-compassion, both strong predictors of employee well-being and performance.[53-55] In line with the JD-R model,[19] we may conclude that equipping physicians with personal resources can be a decisive factor in the prevention of burnout. That is, when physicians expand their personal resources, their ability to impact the environment increases,[18] enhancing the chance that they will feel equipped to face stressful job demands and ultimately preventing burnout.

Strengths and weaknesses

For peer review only - http://bmjopen.bmj.com/site/about/guidelines.xhtml

To our knowledge, our study provides first evidence from a controlled intervention study on the effectiveness of coaching in both medical residents and specialists. Additionally, the two-wave design including a control group together with the additional analyses we conducted allow for a sound interpretation of the intervention effects demonstrating meaningful changes in a group of physicians (in training) who are motivated to accept assistance. However, it should be noted that the current study is limited by its quasi-experimental design. The initial differences between the groups may be the result of appropriate self-selection or may point towards a regression to the mean effect. As such, the implications of our study should be read with care. Second, although our analyses did not suggest that hospital or department affiliation influenced treatment effectiveness greatly, the multisite character of the study including different hospital and department affiliations in the groups limits our study's potential to draw causal conclusion. Third, our study design does not allow long-term inferences of coaching effectiveness. And finally, the coaching group consisted exclusively of pediatric residents and physicians. Consequently, more research is needed that evaluates the effectiveness of coaching in different specialties, allowing broader generalization for coaching effectiveness among healthcare professionals.

Strengths and weaknesses in relation to other studies

Intervention studies in healthcare are scarce. However, a recent study investigating the effects of coaching on physician well-being and distress has found that specialists that received 3.5 hours of coaching by telephone showed a reduction in burnout symptoms and improvements in overall quality of life and resilience.[10] While this study highlights the potential of coaching for specialists, the coaching method is not comparable to face-to-face coaching which makes comparison to our study difficult. Both studies however show that coaching leads to a reduction in burnout symptoms. Importantly, our study adds evidence that coaching improves

For peer review only - http://bmjopen.bmj.com/site/about/guidelines.xhtml

well-being and fosters personal resources among residents too. These results suggest that coaching can be beneficial to healthcare professionals from residency onwards.

Possible explanations and implications

Our study provides initial evidence that coaching may also function as a preventive tool through development of personal resources rather than a cure only. It also shows that only six individual coaching sessions, can reduce burnout symptoms. We therefore hope that our results inspire healthcare practitioners and policy makers to prioritize prevention rather than symptom alleviation. While collective action is sorely needed to bring changes on a system level, interventions like coaching can empower the whole spectrum of healthcare professionals from residents onwards to impact the healthcare system and eventually improve quality of care.

Unanswered questions and future research

This study shows that professional coaching can reduce burnout symptoms and strengthen personal resources. However, it is unclear how robust these effects are over time, and if effects can be generalized across different medical specialties. Additionally, the working mechanisms of coaching are yet to be discovered, making these important inquiries for the future.

For peer review only

Acknowledgments

The authors wish to thank all the medical residents and attending physicians as well as the coaches who participated in this study. The authors also want to thank Axel Willemse, Sarah van den Hee, dr. Machteld van den Heuvel, and dr. Angelique Bakker-Pieper for their contribution to the design and implementation of the study.

Contributors

All authors made substantial contributions to the conception and design, and the collection and interpretation of the data. LS, AEMvV, and JK analyzed the data and interpreted the data together with TT, APJdP and MdH. All authors reviewed and approved the final manuscript.

Funding/Support

This research received no specific grant from any funding agency in the public, commercial or not-for-profit sectors.

Competing interests

None declared.

Ethical approval

The institutional Ethics Review Board of the University of Amsterdam gave ethical approval for this study, on December 12, 2016; document 2016-WOP-7521.

Disclaimer

None reported.

Data sharing statement

For peer review only

Data and statistical code are available upon request. Any queries should be directed to the first author.

Previous presentations

This research has been presented at the following international conferences:

Coaching in Leadership and Healthcare Conference, Institute of Coaching, Boston, United States (September 29, 2018)

International Association for Medical Education AMEE Conference, Vienna, Austria (August 26, 2019)

For peer review only

For peer review only - http://bmjopen.bmj.com/site/about/guidelines.xhtml

References

1. Thomas NK. Resident burnout. JAMA. 2004; 292:2880-2889.
2. Shanafelt TD, Hasan O, Dyrbye LN, et al. Changes in burnout and satisfaction with work-life balance in physicians and the general US working population between 2011 and 2014. Mayo Clin Proc. 2015; 90:1600-1613.
3. Shanafelt TD, Sloan JA, Habermann TM. The well-being of physicians. Am J Med. 2003; 114:513-519.
4. Hall LH, Johnson J, Watt I, Tsipa A, O'Connor DB. Healthcare staff wellbeing, burnout, and patient safety: a systematic review. PLoS One. 2016; 11, e0159015
5. Salyers MP, Bonfils KA, Luther L, et al. The relationship between professional burnout and quality and safety in healthcare: a meta-analysis. J Gen Intern Med. 2017; 32, 475-482
6. Grant AM. The impact of life coaching on goal attainment, metacognition and mental health. Soc Behav Personal. 2003; 31:253-263.
7. Theeboom T, Van Vianen AE, Beersma B. A temporal map of coaching. Front Psychol. 2017; 8:1352
8. Theeboom T. *Workplace coaching: Processes and effects*. [PhD thesis]. Amsterdam, The Netherlands: University of Amsterdam;2016
9. Noe RA. An investigation of the determinants of successful assigned mentoring relationships. Pers Psychol. 1988; 41:457-479.
10. Dyrbye LN, Shanafelt TD, Gill PR, et al. Effect of a professional coaching intervention on the well-being and distress of physicians: A pilot randomized clinical trial. JAMA Intern Med. 2019; 179:1406-1414.
11. Firth-Cozens J. Interventions to improve physicians' well-being and patient care. Soc Sci Med. 2001; 52:215-222.

For peer review only - http://bmjopen.bmj.com/site/about/guidelines.xhtml

12. Firth-Cozens J. Doctors their wellbeing, and their stress. It's time to be proactive about stress–and to prevent it. BMJ. 2003; 326:670-671.

13. Manek N. Coaching for doctors: a road to salvation. BMJ. 2004; 329(7458), s29-s29.

14. Lovell B. What do we know about coaching in medical education? A literature review. Med Educ. 2018; 52:376-390.

15. Theeboom T, Beersma B, van Vianen AE. Does coaching work? A meta-analysis on the effects of coaching on individual level outcomes in an organizational context. The J Pos Psychol. 2014; 9:1-18.

16. Biswas-Diener R. Personal coaching as a positive intervention. J Clin Psychol. 2009; 65:544-553.

17. Demerouti E, Bakker AB, Nachreiner F, Schaufeli WB. The job demands-resources model of burnout. J Appl Psychol. 2001; 86:499-512.

18. Hobfoll SE, Johnson RJ, Ennis N, Jackson AP. Resources loss, resource gain, and emotional outcomes among inner city women. J Pers Soc Psychol 2003;84:632-643.

19. Xanthopoulou D, Bakker AB, Demerouti E, Schaufeli WB. The role of personal resources in the job demands-resources model. Int J Stress Manag. 2007; 14:121-141.

20. Cook TD, Campbell DT. The design and conduct of true experiments and quasi-experiments in field settings. In Mowday RT, Steers RM, eds. Reproduced in part in *Research in Organizations: Issues and Controversies*. Santa Monica, CA: Goodyear Publishing Company; 1979.

21. McKenna DD, Davis SL. Hidden in plain sight: The active ingredients of executive coaching. Ind Organ Psychol. 2009; *2*:244-260.

22. Bordin ES. The generalizability of the psychoanalytic concept of the working alliance. Psychother -Theor Res. 1979; 16:252-260.

For peer review only - http://bmjopen.bmj.com/site/about/guidelines.xhtml

23. Spector PE, Jex SM. Development of four self-report measures of job stressors and strain: interpersonal conflict at work scale, organizational constraints scale, quantitative workload inventory, and physical symptoms inventory. J Occup Health Psychol. 1998; 3:356-367.

24. De Witte H. Arbeidsethos en jobonzekerheid: Meting en gevolgen voor welzijn, tevredenheid en inzet op het werk [Work ethics and job insecurity: Measurement and consequences for well-being, satisfaction, and work engagement]. In: Bouwen R, De Witte K, De Witte H, Taillieu T. Van groep naar gemeenschap. Liber Amicorum Prof. Dr. Leo Lagrou. Leuven, Belgium: Garant. 2000:325-350.

25. Netemeyer RG, Boles JS, McMurrian R. Development and validation of work–family conflict and family–work conflict scales. J Appl Psychol. 1996; 81:400-410.

26. Morgeson FP, Humphrey SE. The Work Design Questionnaire (WDQ): developing and validating a comprehensive measure for assessing job design and the nature of work. J Appl Psychol. 2006; 91:1321-1329.

27. Vinokur A, Schul Y, Caplan RD. Determinants of perceived social support: Interpersonal transactions, personal outlook, and transient affective states. J Pers Soc Psychol. 1987; 53:1137-1145.

28. Luthans F, Youssef CM, Avolio BJ. Psychological capital: Developing the human competitive edge. Oxford: Oxford University Press; 2007.

29. Schwarzer R, Jerusalem M. Generalized self-efficacy scale. In Weinman J, Wright S, Johnston M, eds. Measures in Health Psychology: A User's Portfolio. Causal and Control Beliefs. Windsor, UK: NFER-NELSON; 1995:35-37.

30. Vink J, Ouweneel E, Le Blanc P. Psychologische energiebronnen voor bevlogen werknemers: Psychologisch kapitaal in het Job Demands-Resources model. Gedrag en Organisatie. 2011; 24:101-120.

For peer review only - http://bmjopen.bmj.com/site/about/guidelines.xhtml

31. Neff KD, Vonk R. Self-compassion versus global self-esteem: Two different ways of relating to oneself. J Pers. 2009; 77:23-50.

32. Bond FW, Lloyd J, Guenole N. The work-related acceptance and action questionnaire: Initial psychometric findings and their implications for measuring psychological flexibility in specific contexts. J Occup Organ Psychol. 2013; 86:331-347.

33. Schaufeli WB, van Dierendonck D. UBOS Utrechtse Burnout Schaal: Handleiding. Lisse, The Netherlands: Swets Test Publishers; 2000.

34. Maslach C, Jackson SE, Leiter MP, Schaufeli WB, Schwab RL. Maslach burnout inventory. Palo Alto, CA: Consulting Psychologists Press; 1986; 21:3463-3464.

35. Schaufeli WB, Martinez IM, Pinto AM, Salanova M, Bakker AB. Burnout and engagement in university students: A cross-national study. J Cross Cult Psychol. 2002; 33:464-481.

36. Rø KEI, Gude T, Tyssen R, Aasland OG. Counselling for burnout in Norwegian doctors: one year cohort study. BMJ. 2008; 337:a2004.

37. Van Breukelen GJ. ANCOVA versus change from baseline had more power in randomized studies and more bias in nonrandomized studies. J Clin Epidemiol. 2006; 59:920-925.

38. Huberty CJ, Morris JD. Multivariate analysis versus multiple univariate analyses. Psychol Bull. 1989; 105:302-308.

39. Van Vianen, A.E.M. Person-Organization Fit: The match between newcomers' and recruiters' preferences for organizational cultures. Pers Psychol. 2000; 53:113-149.

40. Edmondson A. Psychological safety and learning behavior in work teams. Adm Sci Q. 1999; 44:350-383.

For peer review only - http://bmjopen.bmj.com/site/about/guidelines.xhtml

41. Van Dyck C, Frese M, Baer M, Sonnentag S. Organizational error management culture and its impact on performance: a two-study replication. J Appl Psychol. 2005; 90:1228-1240.

42. Lemaire J, Wallace JE. Burnout among doctors. BMJ (Online). 2017; 358.

43. Wright AA, Katz IT. Beyond burnout - redesigning care to restore meaning and sanity for physicians. New Engl J Med. 2018; 378:309-311.

44. Dzau VJ, Kirch DG, Nasca TJ. To care is human—collectively confronting the clinician-burnout crisis. New Engl J Med. 2018; 378:312-314.

45. Eisenstein L. To fight burnout, organize. New Engl J Med. 2018; 379:509-511.

46. Lovell B. What do we know about coaching in medical education? A literature review. Med Edu. 2018, 52:376-390.

47. Green LS, Oades LG, Grant AM. Cognitive-behavioral, solution-focused life coaching: Enhancing goal striving, well-being, and hope. J Posit Psychol. 2006; 1:142-149.

48. Grant AM, Green LS, Rynsaardt J. Developmental coaching for high school teachers: Executive coaching goes to school. Consult Psychol J: Pract Res. 2010; 62:151-168.

49. Ireland MJ, Clough B, Gill K, Langan F, O'Connor A, Spencer L. A randomized controlled trial of mindfulness to reduce stress and burnout among intern medical practitioners. Med Teach. 2017; 39:409-414.

50. Wright B, Mynett JR. Training medical students to manage difficult circumstances-a curriculum for resilience and resourcefulness? BMC Med Educ. 2019; 19:280.

51. Panagioti M, Panagopoulou E, Bower P, et al. Controlled interventions to reduce burnout in physicians: a systematic review and meta-analysis. JAMA Intern Med. 2017; 177:195-205.

For peer review only - http://bmjopen.bmj.com/site/about/guidelines.xhtml

52. Maslach C, Schaufeli WB, Leiter MP. Job burnout. Annu Rev Psychol. 2001; 52:397-422.

53. Luthans F, Avolio BJ, Avey JB, Norman SM. Positive psychological capital: Measurement and relationship with performance and satisfaction. Pers Psychol. 2007; 60:541-572.

54. Avey JB, Luthans F, Smith RM, Palmer NF. Impact of positive psychological capital on employee well-being over time. J Occu Health Psychol 2010; 15:17-28.

55. Neff KD. The role of self-compassion in development: A healthier way to relate to oneself. Hum Dev. 2009; 52:211-214.

For peer review only

Figure Titles

Figure 1. Flow chart of Study Inclusion for Participants in Coaching and Control Group in a Study on Coaching Effectiveness for Medical Residents and Specialists

Figure 2. Graphic Representation of the Outcomes at Baseline and Follow-up Measurement for the Coaching Group and the Control Group in a Study on Coaching Effectiveness for Medical Residents and Specialists

For peer review only

Figure 1. Flow Chart of Study Inclusion for Participants in Coaching and Control Group in a Study on Coaching Effectiveness for Medical Residents and Specialists

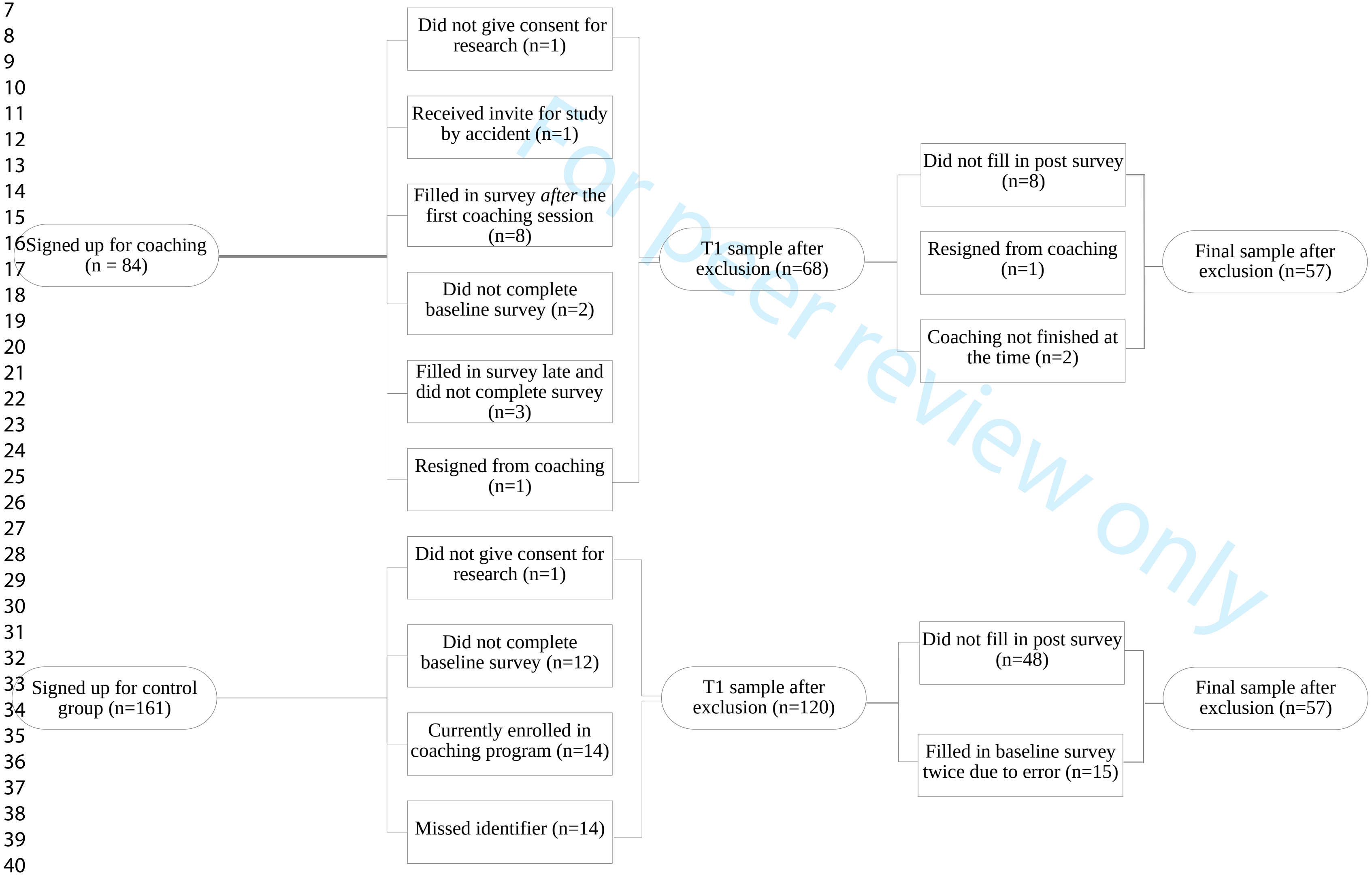

For peer review only

Figure 2. Graphic Representation of the Outcomes at Baseline and Follow-up Measurement for the Coaching Group and the Control Group in a Study on Coaching Effectiveness for Medical Residents and Specialists

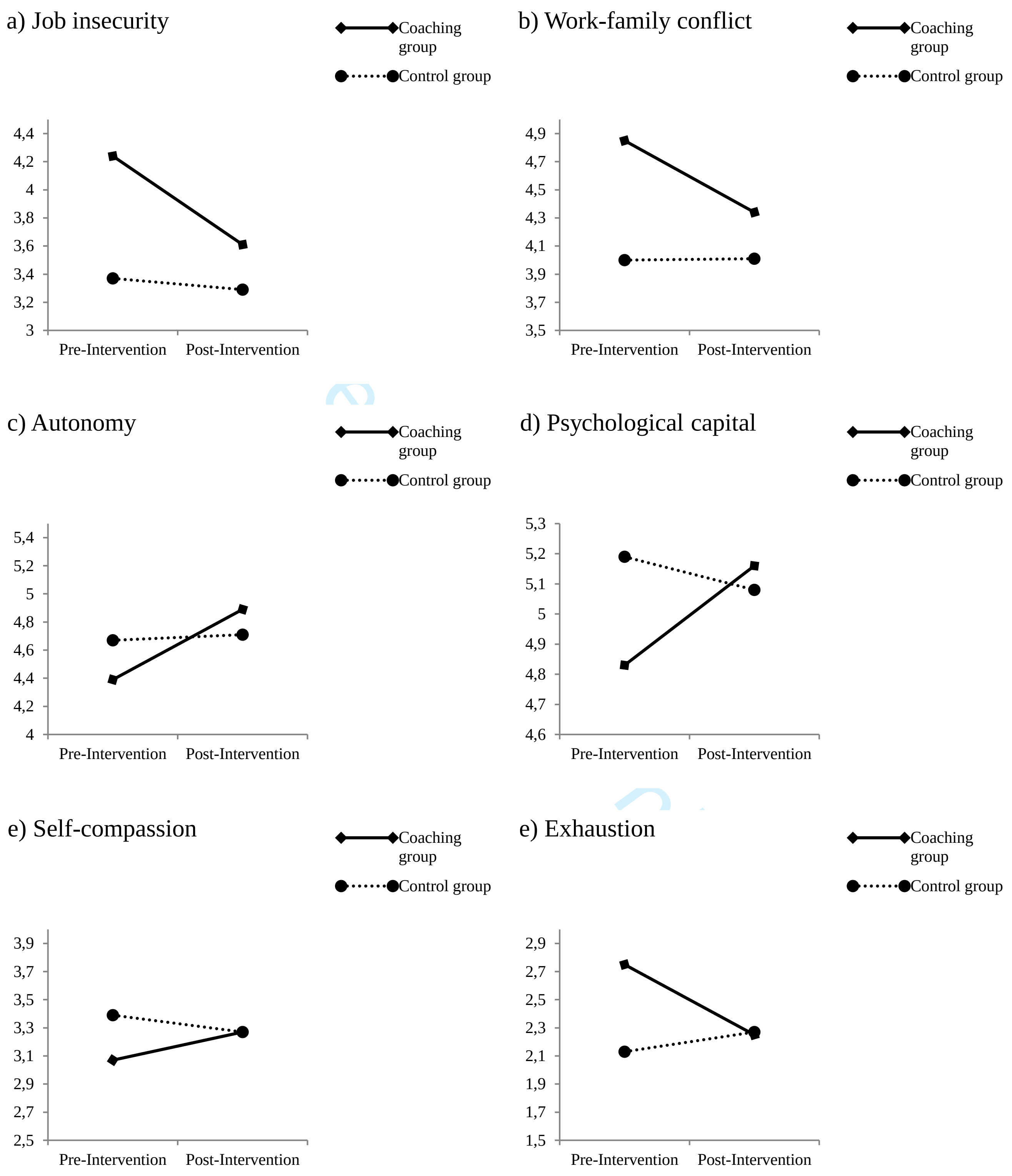

STROBE 2007 (v4) checklist of items to be included in reports of observational studies in epidemiology*
Checklist for cohort, case-control, and cross-sectional studies (combined)

Section/Topic	Item #	Recommendation	Reported on page #
**Title and abstract**	1	(*a*) Indicate the study's design with a commonly used term in the title or the abstract	1
		(*b*) Provide in the abstract an informative and balanced summary of what was done and what was found	3-4
**Introduction**			
Background/rationale	2	Explain the scientific background and rationale for the investigation being reported	6-7
Objectives	3	State specific objectives, including any pre-specified hypotheses	7
**Methods**			
Study design	4	Present key elements of study design early in the paper	7-8
Setting	5	Describe the setting, locations, and relevant dates, including periods of recruitment, exposure, follow-up, and data collection	7-8
Participants	6	(*a*) *Cohort study*—Give the eligibility criteria, and the sources and methods of selection of participants. Describe methods of follow-up<br>*Case-control study*—Give the eligibility criteria, and the sources and methods of case ascertainment and control selection. Give the rationale for the choice of cases and controls<br>*Cross-sectional study*—Give the eligibility criteria, and the sources and methods of selection of participants	7-8, 12-13 (Table 1), Figure 1
		(*b*) *Cohort study*—For matched studies, give matching criteria and number of exposed and unexposed<br>*Case-control study*—For matched studies, give matching criteria and the number of controls per case	-
Variables	7	Clearly define all outcomes, exposures, predictors, potential confounders, and effect modifiers. Give diagnostic criteria, if applicable	8-11
Data sources/ measurement	8*	For each variable of interest, give sources of data and details of methods of assessment (measurement). Describe comparability of assessment methods if there is more than one group	8-11
Bias	9	Describe any efforts to address potential sources of bias	11-12
Study size	10	Explain how the study size was arrived at	8, Figure 1
Quantitative variables	11	Explain how quantitative variables were handled in the analyses. If applicable, describe which groupings were chosen and why	11-13
Statistical methods	12	(*a*) Describe all statistical methods, including those used to control for confounding	11-14
		(*b*) Describe any methods used to examine subgroups and interactions	11-13
		(*c*) Explain how missing data were addressed	8, Figure 1
		(*d*) *Cohort study*—If applicable, explain how loss to follow-up was addressed<br>*Case-control study*—If applicable, explain how matching of cases and controls was addressed	8, Figure 1

For peer review only

		Cross-sectional study—If applicable, describe analytical methods taking account of sampling strategy	
		(*e*) Describe any sensitivity analyses	-
**Results**			
Participants	13*	(a) Report numbers of individuals at each stage of study—eg numbers potentially eligible, examined for eligibility, confirmed eligible, included in the study, completing follow-up, and analysed	14, Figure 1
		(b) Give reasons for non-participation at each stage	Figure 1
		(c) Consider use of a flow diagram	Figure 1
Descriptive data	14*	(a) Give characteristics of study participants (eg demographic, clinical, social) and information on exposures and potential confounders	12-13 (Table 1), 14
		(b) Indicate number of participants with missing data for each variable of interest	-
		(c) *Cohort study*—Summarise follow-up time (eg, average and total amount)	8
Outcome data	15*	*Cohort study*—Report numbers of outcome events or summary measures over time	15-16, Figure 2
		Case-control study—Report numbers in each exposure category, or summary measures of exposure	
		Cross-sectional study—Report numbers of outcome events or summary measures	
Main results	16	(*a*) Give unadjusted estimates and, if applicable, confounder-adjusted estimates and their precision (eg, 95% confidence interval). Make clear which confounders were adjusted for and why they were included	14-17
		(*b*) Report category boundaries when continuous variables were categorized	-
		(*c*) If relevant, consider translating estimates of relative risk into absolute risk for a meaningful time period	-
Other analyses	17	Report other analyses done—eg analyses of subgroups and interactions, and sensitivity analyses	18-19
**Discussion**			
Key results	18	Summarise key results with reference to study objectives	20
Limitations	19	Discuss limitations of the study, taking into account sources of potential bias or imprecision. Discuss both direction and magnitude of any potential bias	21-22
Interpretation	20	Give a cautious overall interpretation of results considering objectives, limitations, multiplicity of analyses, results from similar studies, and other relevant evidence	22
Generalisability	21	Discuss the generalisability (external validity) of the study results	21-22
**Other information**			
Funding	22	Give the source of funding and the role of the funders for the present study and, if applicable, for the original study on which the present article is based	23

*Give information separately for cases and controls in case-control studies and, if applicable, for exposed and unexposed groups in cohort and cross-sectional studies.

Note: An Explanation and Elaboration article discusses each checklist item and gives methodological background and published examples of transparent reporting. The STROBE checklist is best used in conjunction with this article (freely available on the Web sites of PLoS Medicine at http://www.plosmedicine.org/, Annals of Internal Medicine at http://www.annals.org/, and Epidemiology at http://www.epidem.com/). Information on the STROBE Initiative is available at www.strobe-statement.org.

For peer review only
